# HIV, nephrotoxic medications, and chronic kidney disease: Prevalence, risk factors, and mediation analyses among people with and without HIV enrolled in the Multicenter AIDS Cohort Study (MACS)/ Women’s Interagency HIV Study (WIHS) combined cohort study

**DOI:** 10.1371/journal.pone.0336467

**Published:** 2026-06-10

**Authors:** Yue Pan, Dominique L. Musselman, Zain Mithani, Weiqun Tong, Yawen Lu, Joseph B. Margolick, Frank J. Palella, Matthew J. Mimiaga, Kaitlin Bodnar, Deborah Konkle-Parker, Gina Wingood, Daniel Westreich, Eric Seaberg, Signe Lauren, Mardge Cohen, Michelle M. Estrella, Amanda Blair Spence, Tracey Wilson, Michael Ross, Daniel J. Feaster, Maria L. Alcaide, Deborah L. Jones

**Affiliations:** 1 Department of Public Health Sciences, University of Miami, Miami, Florida, United States of America; 2 Department of Psychiatry and Behavioral Sciences, University of Miami, Miami, Florida, United States of America; 3 Division of Nephrology, Department of Medicine, University of Miami Miller School of Medicine, Miami, Florida, United States of America; 4 Department of Epidemiology, Johns Hopkins Bloomberg School of Public Health, Baltimore, Maryland; 5 Department of Molecular Microbiology and Immunology, Johns Hopkins Bloomberg School of Public Health, Baltimore, Maryland; 6 Department of Medicine, Division of Infectious Diseases, Northwestern University Feinberg School of Medicine, Chicago, Illinois, United States of America; 7 Department of Epidemiology, UCLA Fielding School of Public Health and Department of Psychiatry and Biobehavioral Sciences, UCLA Geffen School of Medicine, Los Angeles, California, United States of America; 8 Department of Medicine, Division of Infectious Diseases, University of Pittsburgh, Pittsburgh, Pennsylvania; 9 Schools of Nursing, Medicine and Population Health, University of Mississippi Medical Center, Jackson, Mississippi, United States of America; 10 Department of Sociomedical Sciences, Mailman School of Public Health, Columbia University, New York, New York, United States of America; 11 Department of Epidemiology, Gillings School of Global Public Health, University of North Carolina at Chapel Hill, Chapel Hill, North Carolina; 12 Department of Medicine, Rush University Medical Center; Cook County Health & Hospitals System, Chicago, Illinois, United States of America; 13 Division of Nephrology, Department of Medicine, University of California, San Francisco VA Health Care System, San Francisco, United States of America; 14 Division of Infectious Diseases, Department of Medicine, Georgetown University, Washington, District of Columbia, United States of America; 15 Department of Community Health Sciences, School of Public Health, SUNY Downstate Health Sciences University, Brooklyn, New York, United States of America; 16 Department of Medicine (Nephrology), Albert Einstein College of Medicine, Bronx, New York, United States of America; 17 Department of Medicine, OB/GYN, and Public Health Sciences, University of Miami Miller School of Medicine, Miami, United States of America; Newlands Clinic, ZIMBABWE

## Abstract

**Background:**

Chronic kidney disease (CKD) affects over 37 million adults in the United States, and people living with HIV (PLWH) are at greater risk for progression to end-stage kidney disease. Although both conditions are common among PLWH, the potential pathways through which depression and use of medications with nephrotoxic potential may influence CKD development remain underexplored. We evaluated the relationships of depression and nephrotoxic medication use with CKD prevalence among PLWH, and investigated the potential mediating effects of these factors on the pathway to CKD among PLWH.

**Methods:**

We analyzed data from the Multicenter AIDS Cohort Study (MACS)/Women’s Interagency HIV Study (WIHS) Combined Cohort Study (MWCCS). Baseline CKD prevalence was estimated using Visit 101 only (November 2020-September 2021). To examine associations with CKD over time, we fit generalized estimating equations (GEE) using repeated observations from Visits 101–103 (November 2020-March 30, 2023) with a Poisson distribution and log link to estimate relative risks (RR) and account for within-participant correlation. Counterfactual-based causal mediation analyses were conducted using Visit 101–103 data to evaluate whether elevated depressive symptoms (CES-D ≥ 16) or nephrotoxic medication use mediated the HIV-CKD association while adjusting for baseline confounders.

**Results:**

Among 2,530 participants [1,622 PLWH and 908 people living without HIV (PLWoH)], CKD prevalence was higher in PLWH (18.1%) compared to PLWoH (9.7%). In univariate repeated-measures GEE models, HIV serostatus (RR = 1.37, 95% CI: 1.28–1.48, p < 0.0001) and Nephrotoxic medication use (RR = 1.49, 95% CI: 1.30–1.71, p < 0.0001) were significantly associated with higher CKD risk. Several covariates were also associated with CKD in univariate GEE models, including age (RR = 1.03, 95% CI: 1.03–1.03, p < 0.0001), non-Hispanic Black (RR = 1.19, 95% CI: 1.11–1.27, p < 0.0001) compared to non-Hispanic White, diabetes (RR = 1.26, 95% CI: 1.17–1.35, p < 0.0001), and higher income (RR = 0.99, 95% CI: 0.98–1.00, p = 0.005). Depressive symptoms were not associated with CKD in mediation-model adjusted analyses and did not mediate the HIV-CKD association. Mediation analysis indicated that nephrotoxic medication use accounted for a small but significant proportion of the HIV-CKD association (indirect effect OR = 1.02, 95% CI: 1.00–1.03, p = 0.02).

**Conclusions:**

While it is well established that PLWH have a higher prevalence of CKD compared to PLWoH, our findings suggest that nephrotoxic medication use may modestly amplify this risk. Although most of the risk appears to be attributable to the direct effects of HIV, these medications represent a modifiable contributor. PLWH receiving such treatments may benefit from closer kidney function monitoring. Future research should evaluate psychosocial contributors to CKD using designs that incorporate clinical depression diagnosis and treatment data to clarify depression-related pathways.

## Introduction

People living with HIV (PLWH) enjoy markedly extended survival due to the routine use of virally suppressive antiretroviral therapy. However, PLWH remain at elevated risk for kidney-related complications, including a higher incidence of acute kidney injury and a greater burden of both general (e.g., hypertension and diabetes) and HIV-specific risk factors for chronic kidney disease (CKD). The prevalence of HIV-associated chronic kidney disease (CKD) varies widely by region, patient characteristics, reporting methods, and CKD definition. In North America and Europe, using a definition of eGFR < 60 mL/min/1.73 m² or proteinuria, prevalence may reach 15% or higher [1].

The development of CKD in PLWH is often multifactorial. It may result from HIV-related immune dysfunction (e.g., prolonged immunosuppression, high viral load), genetic predisposition, and the use of certain antiretroviral medications [2]. Compared with the general population, PLWH experience a 2- to 20-fold greater risk of developing end-stage kidney disease [1,3], with a disproportionately higher risk borne by Black individuals [4]. Even more fundamentally, HIV is directly kidney-tropic: in the pre-ART era, HIV-associated nephropathy (HIVAN), characterized by rapid progression to renal failure and collapsing focal segmental glomerulosclerosis was a leading cause of death among Black PLWH [5]. The kidney, as one of HIV’s primary targets, remains a critical focus for renal prevention and therapeutic strategies.

Depression is also common among PLWH, occurring in about 28% of PLWH who are older than 50 years of age [6]. Two recent community-based studies highlighted the relationship between depression and CKD. The China Health and Retirement Longitudinal Study enrolled 4763 participants with eGFR ≥ 60 mL/min per 1.73 m^2^ at baseline and discovered that in persons without kidney disease at baseline, those with depressive symptoms were more likely to show signs of rapid kidney function decline over a median follow-up of 4 years and suffered 39% greater chance of rapid kidney decline, independent of other major demographic, clinical, or psychosocial covariates [7]. The second study highlighted the synergistic impact of depression in persons in the United States (US) who had already developed CKD. The 2022 retrospective analysis of 24,412 participants within the National Health and Nutrition Examination Survey 2005–2014 had a mean follow-up of 5.8 years. Persons with CKD with comorbid depression exhibited over three and a half times the hazard ratio for all-cause and cardiovascular-related mortality, followed by persons with depression without CKD, then persons with CKD only [8]. However, neither study examined the potential role of nephrotoxic medications, nor did they evaluate these relationships in the context of HIV serostatus [9].

Nephrotoxic medications, including antidepressants and antipsychotics, are commonly prescribed to manage depression in PLWH, but some of these medications have known nephrotoxic properties that may exacerbate kidney dysfunction [10]. Nephrotoxic medications may play a mediating role in the relationship between HIV and CKD, either through their nephrotoxic effects or by influencing depression-related pathways that affect kidney function. As PLWH suffer increased rates of depression compared to PLWoH, and depression has been repeatedly associated with increased morbidity and mortality in persons with CKD [11,12], evaluating the impact of depression and nephrotoxic medication usage on kidney function in PLWH is critical.

In this study, we aimed to evaluate the prevalence of CKD at baseline (Visit 101) among PLWH compared to people living without HIV (PLWoH), and to examine whether depression and the use of nephrotoxic medications were independently associated with CKD across repeated study visits (Visits 101–103). We leveraged data from the Multicenter AIDS Cohort Study (MACS)/Women’s Interagency HIV Study (WIHS) Combined Cohort Study (MWCCS), the largest and longest-running prospective cohort of individuals with and without HIV in the United States, to address these questions and inform targeted clinical strategies for kidney health in this population.

Given the high prevalence of depression among PLWH, its known associations with chronic illness outcomes, and the widespread use of nephrotoxic medications, many of which have nephrotoxic potential, it is critical to examine how these factors may contribute to CKD risk in this population. We hypothesized that depression would be associated with increased CKD risk among PLWH. Additionally, we conducted causal mediation analyses to determine whether depressive symptoms and the use of nephrotoxic medications served as mediators in the relationship between HIV serostatus and CKD, thereby identifying potentially modifiable factors along this pathway.

## Materials and methods

### Study design and population

**Fig 1:**
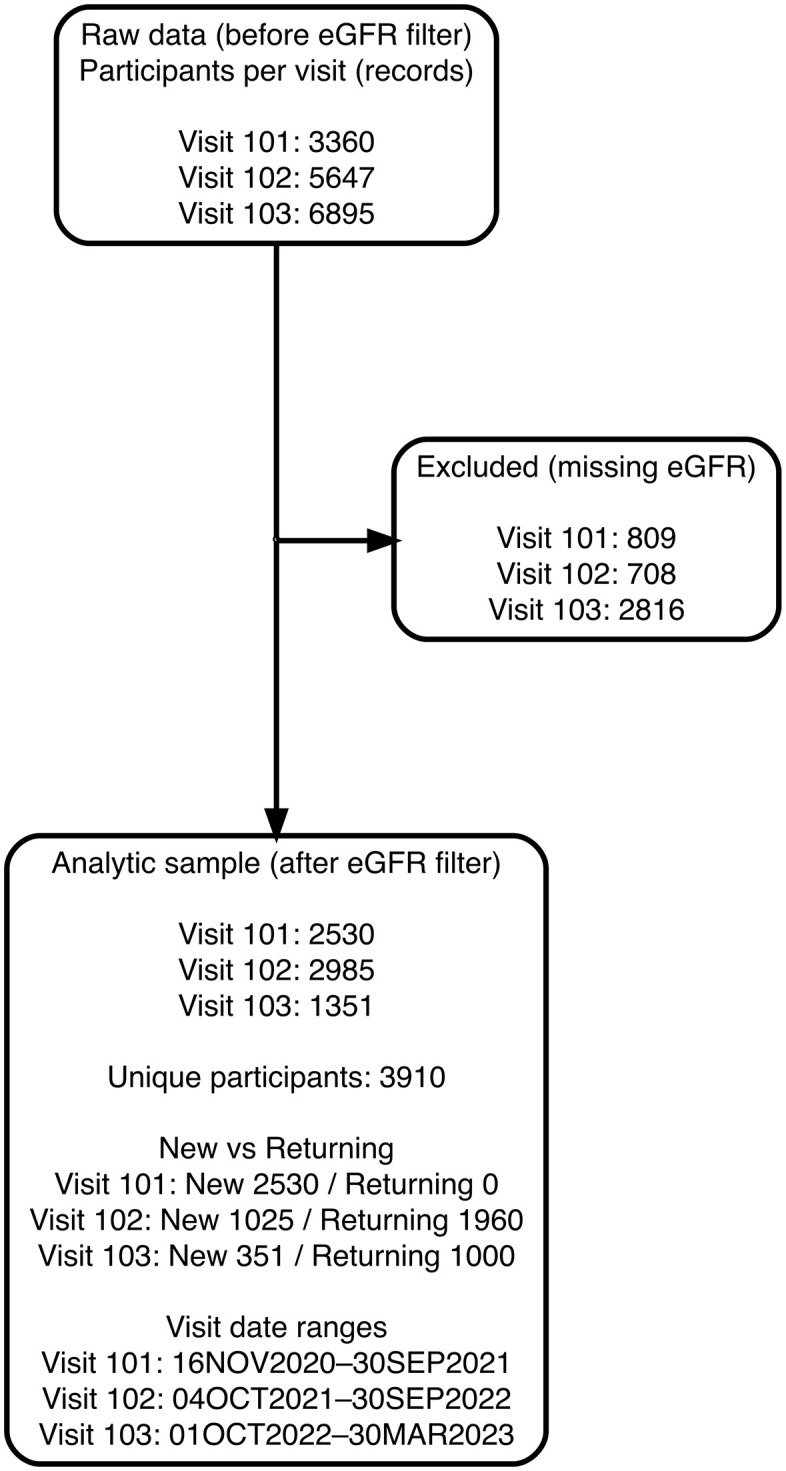
Study flow chart.

The MWCCS is a prospective observational cohort study designed to study the impact of chronic health conditions that affect PLWH in the US. Details have been previously described [13]. For this longitudinal analysis, baseline data were provided by the MWCCS Data Coordinating Center on participants aged 30 and older at the 13 MWCCS sites. Data were derived from three consecutive visits: baseline (Visit 101), follow-up 1 (Visit 102), and follow-up 2 (Visit 103), occurring between November 16, 2020, and March 30, 2023. Visit 101 was selected as “baseline” because it represents the first MWCCS study visit in the consolidated MACS/WIHS cohort with harmonized measures for the current analysis and served as the anchor for both baseline prevalence estimation and repeated-measures modeling. Visit 101 spanned from November 16, 2020, to September 30, 2021; Visit 102 occurred from October 4, 2021, to September 30, 2022; and Visit 103 extended from October 1, 2022, to March 30, 2023. Particularly, we conducted two complementary analyses: (1) a baseline prevalence analysis using Visit 101 only; and (2) a repeated-measures association analysis using Visits 101–103, where CKD status and time-varying covariates were assessed at each visit and correlated within individuals. At Visit 101, 2,530 participants were enrolled as new participants. At Visit 102, 1,030 new participants joined, while 1,958 participants from Visit 101 were retained. By Visit 103, 460 new participants enrolled, and 892 participants from Visit 102 continued their participation. Across all three visits, a total of 6,870 participant records were analyzed, including 4,020 new participants and 2,850 returning participants. Fig 1 presents a flow diagram of participant inclusion for the baseline prevalence analysis (Visit 101) and the repeated-measures GEE analysis (Visits 101–103). All participants provided written informed consent at each site’s institutional review board (IRB)-approved protocol.

This study analyzed baseline demographic, clinical, and behavioral characteristics of participants stratified by HIV serostatus to evaluate the prevalence of CKD. The analysis was conducted from 1,622 PLWH and 908 PLWoH in MWCCS. Variables were categorized and constructed based on participant demographics, socioeconomic factors, clinical diagnoses, behavioral data, and laboratory measurements. The preliminary cross-sectional analyses utilized baseline data from study subjects at visit 101, conducted between October 2020 and September 2021, across all participating MWCCS sites. Demographic data include age, gender, race, and laboratory measures for eGFR calculated using the Chronic Kidney Disease Epidemiology Collaboration (CKD-EPI) equation. Additional covariates at baseline include HIV serostatus, diabetes mellitus, hypertension, cardiovascular disease, body mass index (BMI), and substance use (alcohol, tobacco, injection drug use (IDU), cocaine, opiates). Socioeconomic factors, including education, marital status, and annual household income, were also considered.

### Assessments of kidney function, depressive symptoms, and nephrotoxic medication use

CKD was defined as eGFR < 60 mL/min per 1.73 m2 (using the CKD-EPI equation) at the study visit [14], consistent with the Kidney Disease: Improving Global Outcomes (KDIGO) 2012 clinical practice guidelines for the evaluation and management of CKD. In addition, kidney function was categorized based on estimated glomerular filtration rate (eGFR) into six stages to reflect progressive kidney damage and loss of function. The stages include Kidney Damage with Normal Kidney Function (eGFR ≥ 90 mL/min/1.73 m²), Kidney Damage with Mild Loss of Kidney Function (eGFR ≥ 60 and < 90 mL/min/1.73 m²), Mild to Moderate Loss of Kidney Function (eGFR ≥ 45 and < 60 mL/min/1.73 m²), Moderate to Severe Loss of Kidney Function (eGFR ≥ 30 and < 45 mL/min/1.73 m²), Severe Loss of Kidney Function (eGFR ≥ 15 and < 30 mL/min/1.73 m²), and Kidney Failure (eGFR < 15 mL/min/1.73 m²).

Depression was measured using the Center for Epidemiologic Studies Depression (CES-D) 20-item questionnaire [15]. A cut-off score of ≥16 was used to indicate elevated depressive symptoms, consistent with established screening thresholds applied in both men and women in large cohort studies, including the MACS and WIHS cohorts [16,17].

Nephrotoxic medications were defined as medications with established nephrotoxic properties, based on expert review and retained for analysis in this study. These medications were classified based on their potential nephrotoxic effects and nephrotoxic properties, ensuring clinical relevance in assessing exposure. The medications were assessed at the time of the visit, capturing current medication use for those who may have used them in the past [18]. Medications with nephrotoxic potential were identified using a modified Delphi method, which involved expert consensus across multiple rounds to systematically review and classify medications based on their likelihood of causing kidney toxicity [19]. (Full category list is provided in S2 Table). This list reflects only medications with nephrotoxic properties relevant to CKD risk. Nephrotoxic medication exposure was constructed as a binary indicator at each visit (yes/no), reflecting any recorded use of ≥1 medication on the nephrotoxic list at that visit’s medication inventory. Dose, duration, and cumulative burden were not available in a harmonized form for all agents and were therefore not modeled in the primary analysis. To address heterogeneity, we conducted a prespecified sensitivity analysis using class-specific indicators (S1 and S2 Tables).

### Assessment of covariates

Demographics include self-reported sex at birth, race/ethnicity, and age. Race/ethnicity categories included non-Hispanic white, non-Hispanic Black, non-Hispanic other, and Hispanic. As for socioeconomic indicators, education level (highest level completed) was categorized into: less than high school, completed high school, some college, completed a four-year college degree, and completed graduate school. Marital status was classified into legally/common-law married, living with a partner, widowed, divorced, separated, never married, and other. Employment status included categories of full-time, part-time, not employed, retired, student, and disability. Income was categorized into predefined annual household income brackets. Diabetes and hypertension status were binary indicators derived from MWCCS standardized definitions based on self-reported clinician diagnosis and/or relevant medication use at the visit. Other clinical factors included BMI, categorized into standard weight categories (e.g., underweight, normal weight, overweight, obese). Smoking status was defined as currently smoking (yes/no). Alcohol use was categorized based on binge drinking episodes since the last visit. Drug use included self-reported use of marijuana, cocaine, heroin, or other illicit drugs, as well as injection drug use.

### Statistical analyses

Descriptive statistics were utilized to summarize baseline demographic, clinical, and behavioral characteristics of the participants stratified by HIV serostatus. For continuous variables, means and standard deviations (SD) were calculated, along with medians and ranges. Comparisons between PLWH and PLWoH were assessed using analysis of variance (ANOVA) for continuous variables. For categorical variables, frequencies and percentages were reported, and chi-square tests were employed to evaluate differences between groups.

For the prevalence analysis, we used data from Visit 101 (baseline) only. CKD prevalence was calculated as the proportion of participants meeting the CKD definition (eGFR < 60 mL/min/1.73 m²) within each subgroup of interest. Results were stratified by HIV serostatus (overall, PLWH, and PLWoH) and by demographic, clinical, and behavioral characteristics. All prevalence estimates were calculated using Visit 101 only.

To examine associations between risk factors and CKD, generalized estimating equations (GEE) with a Poisson distribution and log link were used to estimate relative risks while accounting for within-subject correlation across multiple study visits. We report univariate GEE models. Repeated-measures GEE models used Visits 101–103 to account for within-participant correlation over time. Separate univariate models were fitted for each covariate to avoid potential overadjustment and misinterpretation due to collider or mediator bias (i.e., Table 2 fallacy [20]). Accordingly, GEE estimates are interpreted as repeated-measures associations, not causal effects. The analysis included both categorical and continuous covariates, and relative risks (RR) and 95% confidence intervals (CI) were computed for each predictor.

The GEE models incorporated an exchangeable working correlation matrix and accounted for repeated measurements clustered within individuals. Variables examined included HIV serostatus (PLWH vs. PLWoH), age, sex, race/ethnicity, marital status, education level, income, diabetes, hypertension, depression, nephrotoxic medication use, smoking, alcohol use, other substance use, and BMI. For categorical variables with more than two levels (e.g., education, marital status, race/ethnicity), comparisons were made relative to the reference group. Statistical significance was determined using Wald tests, and model fit was evaluated using empirical standard errors and robust variance estimates. Because CKD status, depressive symptoms, nephrotoxic medication exposure, and several covariates were assessed at the same study visit, the GEE models primarily estimate contemporaneous associations and cannot fully resolve bidirectional relationships (e.g., CKD influencing medication choice or depressive symptoms). Reverse causation and confounding by indication are therefore plausible, particularly for medication classes prescribed in response to kidney risk (e.g., ACEi/ARB, diuretics).

**Fig 2:**
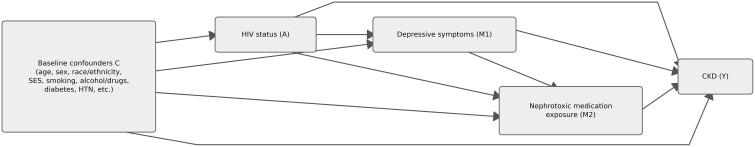
Directed acyclic graph (DAG). Directed acyclic graph (DAG) for the hypothesized pathways linking HIV serostatus (A) to CKD (Y) through depressive symptoms (M1) and nephrotoxic medication exposure (M2), with baseline confounders **(C)**. The DAG encodes potential direct (A → Y) and indirect pathways (A → M1 → Y; A → M2 → Y), allows depressive symptoms to influence nephrotoxic medication exposure (M1 → M2), and represents common causes (C) of A, M1, M2, and **Y.** Confounders in C were included in the mediator and outcome models for the counterfactual mediation analyses.

We prespecified a causal model to guide confounder control for the mediation analyses (Fig 2). In this DAG, HIV serostatus (A) may affect CKD (Y) directly and indirectly through two candidate mediators: elevated depressive symptoms (M1) and nephrotoxic medication exposure (M2). We also allowed a pathway from depressive symptoms to nephrotoxic medication exposure (M1 → M2) to reflect potential prescribing or use patterns related to depressive symptoms. A set of baseline covariates (C) was treated as common causes of HIV serostatus, depressive symptoms, nephrotoxic medication exposure, and CKD, and therefore were included as confounders in the mediator and outcome models. Based on this structure, confounders included age at visit, sex at birth, race/ethnicity, marital status, employment status, diabetes, hypertension, current smoking, binge drinking, marijuana use, cocaine use, heroin use, other drug use, and injection drug use (IDU). Because CKD status and nephrotoxic medication exposure were assessed at the same study visit, reverse causation and confounding by indication remain possible; accordingly, mediation estimates are interpreted under the assumptions encoded by the DAG.

To avoid overadjustment bias, mediators (depression and nephrotoxic medication use) were not included in the total-effect model. The total effect (TE) was first estimated without the inclusion of the mediator. Separate mediator and outcome models were then specified to estimate the natural direct effect (NDE) and natural indirect effect (NIE). Using a binomial model with a logit link, we estimated the total effect (TE), the natural direct effect (NDE), and the natural indirect effect (NIE) on the odds-ratio scale. The NDE represents the effect of HIV on CKD not operating through the mediator, whereas the NIE quantifies the pathway operating through the mediator. Exposure-mediator interaction was formally allowed in the models to permit decomposition under potential effect modification. Bootstrap resampling with 1,000 iterations was used to derive robust 95% confidence intervals for all pathway-specific effects. A two-sided p-value of <0.05 was considered statistically significant.

As a sensitivity analysis regarding the heterogeneity of nephrotoxic medication exposure, we additionally examined class-specific associations between six nephrotoxic drug categories and CKD. Medications were classified into the following groups based on pharmacological mechanism and established nephrotoxic potential: (1) NSAIDs and analgesics (e.g., ibuprofen, naproxen, celecoxib); (2) ACE inhibitors and ARBs (e.g., lisinopril, losartan, olmesartan); (3) diuretics (e.g., furosemide, hydrochlorothiazide, spironolactone); (4) nephrotoxic antiretrovirals, primarily tenofovir-containing regimens (e.g., Truvada, Biktarvy, Genvoya); (5) nephrotoxic antimicrobials (e.g., trimethoprim-sulfamethoxazole, ciprofloxacin, vancomycin, acyclovir); and (6) other nephrotoxic medications (e.g., metformin, lithium, gabapentin, proton pump inhibitors). Binary indicators were created for each class at the person-visit level among participants with visits 101, 102, or 103 and non-missing eGFR. Separate GEE models (Poisson log-link, exchangeable correlation structure) were fit for each class as the primary analysis. Results are presented in S2 Table.

## Results

### Study participants and baseline characteristics

Fig 1 summarizes the construction of the baseline analytic sample and the repeated-measures dataset (Visits 101–103). In the raw data, the number of unique participants contributing data at each visit was 3,339 (Visit 101), 3,693 (Visit 102), and 4,167 (Visit 103). Participants with missing eGFR were excluded at each visit (Visit 101: n = 828; Visit 102: n = 1,289; Visit 103: n = 3,908). After applying the eGFR completeness filter, the analytic sample included 2,530 participants at Visit 101, 2,985 participants at Visit 102, and 1,351 participants at Visit 103, with 3,910 unique participants contributing at least one eligible visit across Visits 101–103.

With respect to cohort dynamics, all eligible Visit 101 participants were new enrollees (Visit 101: New = 2,530; Returning = 0). At Visit 102, 1,028 were new participants and 1,957 were returning from Visit 101. At Visit 103, 352 were new participants and 999 were returning from Visit 102. The eligible visit date ranges were: Visit 101 (16NOV2020−30SEP2021), Visit 102 (04OCT2021−30SEP2022), and Visit 103 (01OCT2022−30MAR2023).

The baseline prevalence analysis (Visit 101) included 1,622 PLWH and 908 PLWoH participants (Table 1). PLWH had a slightly younger mean age (54.7 years) compared to PLWoH (56.0 years, p = 0.002). A higher proportion of PLWH were female (73.4%) compared to PLWoH (57.5%, p < 0.0001). The majority of participants were non-Hispanic Black individuals (66.0% of PLWH and 54.1% of PLWoH). Socioeconomic indicators revealed disparities in education and income levels, with PLWH more likely to have lower educational attainment and income levels than PLWoH (p < 0.0001).

**Table 1:** Baseline Demographic, Clinical, and Behavioral Characteristics of Participants by HIV Status.

	PLWH (N = 1622)	PLWoH (N = 908)	P-value
**Chronic Kidney Disease**			<0.0001
No	1328 (81.9%)	820 (90.3%)	
Yes	294 (18.1%)	88 (9.7%)	
**Participant’s Age at Visit**			0.002
Mean (SD)	54.7 (9.81)	56.0 (11.7)	
Median [Min, Max]	56.0 [29.0, 85.0]	57.0 [30.0, 84.0]	
**Sex at Birth**			<0.0001
Female	1190 (73.4%)	522 (57.5%)	
Male	432 (26.6%)	386 (42.5%)	
**Race/Ethnicity**			<0.0001
Non-Hispanic White	297 (18.3%)	275 (30.3%)	
Non-Hispanic Black	1070 (66.0%)	491 (54.1%)	
Non-Hispanic Other	62 (3.8%)	32 (3.5%)	
Hispanic	193 (11.9%)	110 (12.1%)	
**Education**			<0.0001
Less than high school	428 (26.4%)	195 (21.5%)	
Completed high school	452 (27.9%)	190 (21.0%)	
Some college	438 (27.0%)	228 (25.2%)	
Completed four-year college degree	162 (10.0%)	120 (13.2%)	
Attended/Completed graduate school	142 (8.8%)	173 (19.1%)	
**Marital Status**			<0.0001
Legally/Common-law Married	322 (20.3%)	223 (25.2%)	
Not Married but Living with Partner	116 (7.3%)	102 (11.5%)	
Widowed	134 (8.4%)	45 (5.1%)	
Divorced	201 (12.7%)	90 (10.2%)	
Separated	83 (5.2%)	54 (6.1%)	
Never Married	626 (39.5%)	321 (36.2%)	
Other	104 (6.6%)	51 (5.8%)	
**Employment Status**			<0.0001
Disability	560 (35.4%)	189 (21.4%)	
Employed Full-Time	442 (27.9%)	274 (31.0%)	
Employed Part-Time	156 (9.8%)	86 (9.7%)	
Not Employed	268 (16.9%)	170 (19.2%)	
Retired	149 (9.4%)	160 (18.1%)	
Student	9 (0.6%)	6 (0.7%)	
**Income**			<0.0001
$6000 or less	109 (7.1%)	73 (8.6%)	
$6001-$12,000	470 (30.6%)	173 (20.4%)	
$12,001-$18,000	214 (13.9%)	96 (11.3%)	
$18,001-$24,000	139 (9.1%)	59 (6.9%)	
$24,001-$30,000	110 (7.2%)	56 (6.6%)	
$30,001-$36,000	85 (5.5%)	55 (6.5%)	
$36,001-$75,000	233 (15.2%)	161 (18.9%)	
$75,001-$100,000	57 (3.7%)	55 (6.5%)	
$100,001-$150,000	64 (4.2%)	62 (7.3%)	
$150,001-$200,000	30 (2.0%)	26 (3.1%)	
More than $200,000	24 (1.6%)	34 (4.0%)	
**Race free estimated Glomerular Filtration Rate (mL/min/1.73 m**^**2**^)			<0.0001
Mean (SD)	78.6 (21.1)	86.2 (19.5)	
Median [Min, Max]	79.7 [5.10, 127]	88.5 [4.93, 135]	
**Kidney Function (eGFR)**			<0.0001
Normal Kidney Function (≥90)	507 (31.3%)	420 (46.3%)	
Mild Loss of Kidney Function (≥60 and <90)	821 (50.6%)	400 (44.1%)	
Mild to Moderate Loss of Kidney Function (≥45 and <60)	202 (12.5%)	56 (6.2%)	
Moderate to Severe Loss of Kidney Function (≥30 and <45)	54 (3.3%)	21 (2.3%)	
Severe Loss of Kidney Function (≥15 and <30)	23 (1.4%)	8 (0.9%)	
Kidney Failure (<15)	15 (0.9%)	3 (0.3%)	
**Body Mass Index (kg/m²)**			0.702
Mean (SD)	31.7 (8.74)	31.6 (8.26)	
Median [Min, Max]	30.0 [12.9, 75.6]	30.0 [14.1, 67.8]	
**Has Diabetes**			0.253
No	1224 (75.6%)	704 (77.6%)	
Yes	395 (24.4%)	203 (22.4%)	
**Hypertension Status**			0.156
No	833 (51.4%)	493 (54.3%)	
Yes	789 (48.6%)	415 (45.7%)	
**Nephrotoxic Medication Use**			0.3771
No	751 (46.3%)	437 (48.1%)	
Yes	871 (53.7%)	471 (51.9%)	
**Has Depression**			0.485
No	1096 (72.5%)	609 (71.1%)	
Yes	416 (27.5%)	247 (28.9%)	
**CESD**			0.784
Mean (SD)	11.5 (10.9)	11.4 (10.9)	
Median [Min, Max]	8.00 [0, 54.0]	8.00 [0, 52.0]	
**Current Smoking Status**			0.214
Currently smoking	428 (28.0%)	263 (30.4%)	
Not currently smoking	1100 (72.0%)	602 (69.6%)	
**Hazardous Drinking Since Last Visit**			0.002
No	940 (91.7%)	502 (86.9%)	
Yes	85 (8.3%)	76 (13.1%)	
**Binge Drinking Since Last Visit**			0.157
No	1222 (75.3%)	653 (71.9%)	
Yes	246 (15.2%)	153 (16.9%)	
Missing	154 (9.5%)	102 (11.2%)	
**Used Marijuana Since Last Visit**			0.004
No	1067 (65.8%)	550 (60.6%)	
Yes	460 (28.4%)	314 (34.6%)	
Missing	95 (5.9%)	44 (4.8%)	
**Cocaine Use Since Last Visit**			0.018
No	1405 (86.6%)	766 (84.4%)	
Yes	120 (7.4%)	96 (10.6%)	
Missing	97 (6.0%)	46 (5.1%)	
**Heroin Use Since Last Visit**			<0.0001
No	1503 (92.7%)	827 (91.1%)	
Yes	21 (1.3%)	36 (4.0%)	
Missing	98 (6.0%)	45 (5.0%)	
**Other Drug Use Since Last Visit**			0.04
No	1354 (83.5%)	740 (81.5%)	
Yes	171 (10.5%)	124 (13.7%)	
Missing	97 (6.0%)	44 (4.8%)	
**Injection Drug Use Since Last Visit**			0.373
No	1511 (93.2%)	853 (93.9%)	
Yes	16 (1.0%)	12 (1.3%)	
Missing	95 (5.9%)	43 (4.7%)	

Significant differences were observed in the prevalence of CKD, with 18.1% of PLWH affected compared to 9.7% of PLWoH participants (p < 0.0001). Additionally, measures of kidney function indicated worse outcomes among the PLWH, with fewer participants retaining normal kidney function (31.3% vs. 46.3%, p < 0.0001).

### Prevalence of CKD overall and stratified by HIV serostatus

Table 2 presents CKD prevalence across demographic, clinical, and behavioral characteristics, stratified by HIV serostatus. Higher CKD prevalence was observed among females, individuals with lower educational attainment, and persons identifying as non-Hispanic Black or non-Hispanic Other. Among marital categories, widowed and participants in the “other” marital status category exhibited elevated CKD rates. Participants who were retired or had a disability showed higher CKD prevalence compared to those employed full-time. Behavioral factors such as current smoking, binge drinking, and illicit drug use were associated with higher CKD prevalence, particularly among PLWH.

**Table 2:** Prevalence of chronic kidney disease by participant characteristics, overall and stratified by hiv status.

		Overall		PLWH		PLWoH	
		N	Prevalence (%)	N	Prevalence (%)	N	Prevalence (%)
**HIV serostatus at time of interview**	HIV seronegative	88/908	9.7	–	–	–	–
	HIV positive	294/1622	18.1	–	–	–	–
**Sex at Birth**	Female	285/1712	16.7	224/1190	18.8	61/522	11.7
	Male	97/818	11.9	70/432	16.2	27/386	7.0
**Race/Ethnicity**	Non-Hispanic White	70/572	12.2	51/297	17.2	19/275	6.9
	Non-Hispanic Black	260/1561	16.7	201/1070	18.8	59/491	12.0
	Non-Hispanic Other	17/94	18.1	15/62	24.2	2/32	6.3
	Hispanic	35/303	11.6	27/193	14.0	8/110	7.3
**Education**	Less than high school	92/623	14.8	67/428	15.7	25/195	12.8
	Completed high school	101/642	15.7	82/452	18.1	19/190	10.0
	Some college	118/666	17.7	93/438	21.2	25/228	11.0
	Completed four-year college degree	40/282	14.2	30/162	18.5	10/120	8.3
	Attended/Completed graduate school	31/315	9.8	22/142	15.5	9/173	5.2
**Marital Status**	Legally/Common-law Married	73/545	13.4	49/322	15.2	24/223	10.8
	Not Married but Living with Partner	24/218	11.0	18/116	15.5	6/102	5.9
	Widowed	50/179	27.9	41/134	30.6	9/45	20.0
	Divorced	50/291	17.2	42/201	20.9	8/90	8.9
	Separated	14/137	10.2	7/83	8.4	7/54	13.0
	Never Married	129/947	13.6	102/626	16.3	27/321	8.4
	Other	33/155	21.3	27/104	26.0	6/51	11.8
**Employment Status**	Not Employed	55/438	12.6	43/268	16.0	12/170	7.1
	Employed Full-Time	57/716	8.0	45/442	10.2	12/274	4.4
	Employed Part-Time	26/242	10.7	21/156	13.5	5/86	5.8
	Retired	71/309	23.0	47/149	31.5	24/160	15.0
	Student	0/15	0.0	0/9	0.0	0/6	0.0
	Disability	163/749	21.8	129/560	23.0	34/189	18.0
**Income**	$6000 or less	26/182	14.3	16/109	14.7	10/73	13.7
	$6001-$12,000	136/643	21.2	106/470	22.6	30/173	17.3
	$12,001-$18,000	60/310	19.4	50/214	23.4	10/96	10.4
	$18,001-$24,000	30/198	15.2	25/139	18.0	5/59	8.5
	$24,001-$30,000	23/166	13.9	19/110	17.3	4/56	7.1
	$30,001-$36,000	15/140	10.7	12/85	14.1	3/55	5.5
	$36,001-$75,000	44/394	11.2	29/233	12.5	15/161	9.3
	$75,001-$100,000	7/112	6.3	5/57	8.8	2/55	3.6
	$100,001-$150,000	10/126	7.9	10/64	15.6	0/62	0.0
	$150,001-$200,000	7/56	12.5	4/30	13.3	3/26	11.5
	More than $200,000	2/58	3.5	1/24	4.2	1/34	2.9
**Has Diabetes**	No	240/1928	12.5	190/1224	15.5	50/704	7.1
	Yes	142/598	23.8	104/395	26.3	38/203	18.7
**Hypertension Status**	No	126/1326	9.5	107/833	12.9	19/493	3.9
	Yes	256/1204	21.3	187/789	23.7	69/415	16.6
**Has Depression**	No	261/1705	15.3	200/1096	18.3	61/609	10.0
	Yes	92/663	13.9	71/416	17.1	21/247	8.5
**Nephrotoxic Medication Use**	No	124/1188	10.4	99/751	13.2	25/437	5.7
	Yes	258/1342	19.2	195/871	22.4	63/471	13.4
**Current Smoking Status**	Not currently smoking	248/1702	14.6	198/1100	18.0	50/602	8.3
	Currently smoking	105/691	15.2	72/428	16.8	33/263	12.6
**Binge Drinking Since Last Visit**	No	294/1875	15.7	229/1222	18.7	65/653	10.0
	Yes	38/399	9.5	29/246	11.8	9/153	5.9
**Used Marijuana Since Last Visit**	No	261/1617	16.1	206/1067	19.3	55/550	10.0
	Yes	92/774	11.9	64/460	13.9	28/314	8.9
**Cocaine Use Since Last Visit**	No	315/2171	14.5	247/1405	17.6	68/766	8.9
	Yes	36/216	16.7	22/120	18.3	14/96	14.6
**Heroin Use Since Last Visit**	No	343/2330	14.7	266/1503	17.7	77/827	9.3
	Yes	9/57	15.8	4/21	19.1	5/36	13.9
**Other Drug Use Since Last Visit**	No	323/2094	15.4	247/1354	18.2	76/740	10.3
	Yes	29/295	9.8	22/171	12.9	7/124	5.7
**Injection Drug Use Since Last Visit**	No	349/2364	14.8	268/1511	17.7	81/853	9.5
	Yes	4/28	14.3	2/16	12.5	2/12	16.7

Depression showed a nuanced association with CKD. Among individuals without depression, CKD prevalence was 15.3%, higher in PLWH (18.3%) than PLWoH (10%). Among those with depression, overall CKD prevalence was 13.9%, also higher in PLWH (17.1%) than PLWoH (8.5%). At Visit 101, 1,342 participants (53%) were classified as using nephrotoxic medications (Table 3). Nephrotoxic medication use was 51.9% in PLWoH and 53.7% in PLWH.

**Table 3:** Prevalence of nephrotoxic medication use by visit and HIV status.

Visit Number	HIV Status	Total Participants	Participants Using Nephrotoxic Medications (n)	Nephrotoxic Medication Use (%)
101	Overall	2,530	1,342	53.0
101	HIV-	908	471	51.9
101	HIV+	1,622	871	53.7
102	Overall	2,985	1,624	54.4
102	HIV-	1,074	566	52.7
102	HIV+	1,911	1,058	55.4
103	Overall	1,351	699	51.7
103	HIV-	440	213	48.4
103	HIV+	911	486	53.4

*Nephrotoxic medication use was defined based on expert review of medications with established nephrotoxic properties (see S1 Table).

### Generalized estimating equations (GEE) analysis of CKD across repeated visits

Table 4 presents univariate repeated-measures associations between participant characteristics and CKD across Visits 101−103. PLWH had a significantly higher risk of kidney function decline compared to PLWoH (RR = 1.37, 95% CI: 1.28–1.48, p < 0.001). Age was also positively associated with poorer kidney function, with each additional year of age associated with a 3% increase in CKD risk (RR = 1.03 per year, 95% CI: 1.03–1.03, p < 0.001).

**Table 4:** GEE analysis results: Factors associated with chronic kidney disease.

Variable	RR	95% CI	P-value
**HIV Serostatus (HIV Positive vs. HIV Negative)**	1.37	(1.28, 1.48)	<0.0001
**Age (per unit increase)**	1.03	(1.03, 1.03)	<0.0001
**Sex (Female vs. Male)**	1.16	(1.08, 1.23)	<0.0001
**Race: Non-Hispanic Black (vs. Non-Hispanic White)**	1.19	(1.11, 1.27)	<0.0001
**Race: Hispanic (vs. Non-Hispanic White)**	2.03	(1.86, 2.21)	<0.0001
**Race: Non-Hispanic Other (vs. Non-Hispanic White)**	2.64	(2.31, 3.02)	<0.0001
**Education: Completed high school (HS) vs. < HS**	0.83	(0.68, 1.03)	0.087
**Education: Some college vs. < HS**	0.87	(0.72, 1.07)	0.184
**Education: Completed/graduate college vs. < HS**	0.81	(0.64, 1.02)	0.069
**Education: Attended/completed post-grad vs. < HS**	0.86	(0.69, 1.06)	0.163
**Marital: Not Married but Living with Partner (vs. Married)**	1.16	(1.01, 1.32)	0.0364
**Marital: Widowed (vs. Married)**	1.16	(0.97, 1.4)	0.1059
**Marital: Divorced (vs. Married)**	1.07	(0.93, 1.25)	0.3432
**Marital: Separated (vs. Married)**	1.16	(1.0, 1.36)	0.0556
**Marital: Never Married (vs. Married)**	1.01	(0.9, 1.12)	0.8762
**Marital: Other (vs. Married)**	1.02	(0.89, 1.18)	0.7433
**Income (per unit increase)**	0.99	(0.98, 1.0)	0.005
**Diabetes (Yes vs. No)**	1.26	(1.17, 1.35)	<0.0001
**Hypertension (Yes vs. No)**	1.06	(0.97, 1.15)	0.2043
**Depression (Yes vs. No)**	1.04	(0.96, 1.14)	0.3355
**Nephrotoxic Medication (Yes vs. No)**	1.49	(1.30, 1.71)	<0.0001
**Current Smoker (vs. Non-Smoker)**	1.09	(0.91, 1.31)	0.3352
**Marijuana Use (Yes vs. No)**	1.1	(0.96, 1.25)	0.1655
**Cocaine Use (Yes vs. No)**	0.92	(0.75, 1.12)	0.3918
**Heroin Use (Yes vs. No)**	0.69	(0.28, 1.71)	0.4257
**Other Drug Use (Yes vs. No)**	0.96	(0.76, 1.21)	0.7075
**Injection Drug Use (Yes vs. No)**	1	(0.97, 1.03)	0.9276
**BMI (per unit increase)**	1.02	(0.99, 1.05)	0.2426

Among racial/ethnic groups, non-Hispanic Black individuals had a significantly higher risk (RR = 1.19, 95% CI: 1.11–1.27, p < 0.001), and Hispanic individuals had more than twice the risk compared to non-Hispanic White individuals (RR = 2.03, 95% CI: 1.86–2.21, p < 0.001). Additionally, non-Hispanic Other individuals also showed an elevated risk (RR = 2.64, 95% CI: 2.31–3.02, p < 0.001). Sex was also significantly associated, with females having higher CKD risk compared to males (RR = 1.16, 95% CI: 1.08–1.23, p < 0.001). Higher income was associated with lower CKD risk (RR = 0.99, 95% CI: 0.98–1.00, p = 0.005). Among chronic conditions, diabetes was significantly associated with increased risk of CKD (RR = 1.26, 95% CI: 1.17–1.35, p < 0.001), while hypertension was not statistically significant (RR = 1.06, 95% CI: 0.97–1.15, p = 0.204).

### Mediation analysis: Depressive symptoms and/or nephrotoxic medication use mediate the relationship between HIV status and CKD

**Fig 3:**
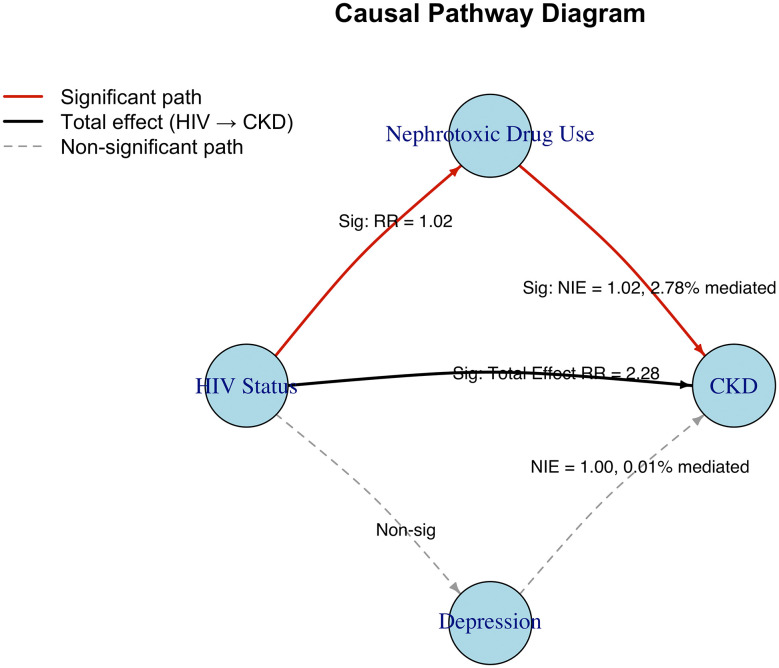
Causal pathway diagram.

We conducted a mediation analysis to evaluate whether depression and nephrotoxic medication use mediated the relationship between HIV status and CKD. The total OR of CKD associated with HIV status was 2.28 (95% CI: 1.88–2.77, p < 0.0001; Fig 3), indicating a strong association between HIV and CKD. The natural direct effect (NDE), i.e., portion of the HIV-CKD association not operating through nephrotoxic medication use, was similar in magnitude (OR = 2.25, 95% CI: 1.84–2.73, p < 0.0001).

The natural indirect effect (NIE) through nephrotoxic medication use was modest but statistically significant (OR = 1.02, 95% CI: 1.00–1.03, p = 0.019), accounting for ~2.78% of the total effect. Most of the association was explained by the direct pathway, with a small yet meaningful portion mediated through nephrotoxic drug exposure. Importantly, the interaction between HIV and nephrotoxic medication use was statistically significant (17.83%, p = 0.0002), suggesting that nephrotoxic drug exposure further increased CKD risk among people living with HIV.

### Sensitivity analysis: Nephrotoxic medication exposure by drug class

Class-specific GEE analyses revealed heterogeneity in the associations between individual nephrotoxic drug classes and CKD among 6,866 person-visits (6,256 with complete covariate data) across visits 101–103 (S2 Table). Exposure prevalence varied substantially across classes: NSAIDs/analgesics (n = 1,136; 16.55%), other medications (n = 1,014; 14.77%), ACE inhibitors/ARBs (n = 535; 7.79%), diuretics (n = 498; 7.25%), antimicrobials (n = 334; 4.86%), and nephrotoxic antiretrovirals (n = 149; 2.17%). ACE inhibitors and ARBs were associated with a significantly elevated CKD risk (RR = 1.32, 95% CI: 1.12–1.56, p = 0.0008). Diuretics showed the strongest association with CKD (RR = 1.49, 95% CI: 1.25–1.77, p < 0.0001). These associations likely reflect confounding by indication, as these drug classes are preferentially prescribed to patients with hypertension, proteinuria, or early kidney disease – the very conditions that predispose to CKD progression. NSAIDs/analgesics (RR = 0.98, 95% CI: 0.86–1.11, p = 0.73) and antimicrobials (RR = 0.87, 95% CI: 0.64–1.18, p = 0.36) showed no statistically significant associations with CKD. Other nephrotoxic medications showed a borderline-significant inverse association (RR = 0.86, 95% CI: 0.75–1.00, p = 0.044), which similarly reflects confounding given that several agents in this heterogeneous class (e.g., metformin, proton pump inhibitors) are used in populations with different underlying risk profiles. Nephrotoxic antiretrovirals showed a non-significant elevated risk (RR = 1.44, 95% CI: 0.91–2.27, p = 0.12); this estimate should be interpreted with caution given only 11 CKD events among 149 exposed person-visits. Taken together, these class-specific findings underscore the heterogeneity of the nephrotoxic medication exposure construct and support the interpretation that the mediation pathway identified in the primary analysis is likely driven by a subset of higher-risk drug classes, particularly those with direct renal hemodynamic or tubular effects.

## Discussion

While prior studies have suggested a link between depression and CKD progression [21,22], our analyses did not identify an independent association between elevated depressive symptoms (CES-D ≥ 16) and CKD after adjustment, nor evidence that depressive symptoms mediated the HIV-CKD association. Because depressive symptoms were measured using CES-D scores without information on clinical diagnosis, antidepressant prescribing, adherence, or treatment response, we cannot determine whether treatment or symptom management influenced these findings; any explanation involving “effective treatment” remains speculative. In contrast, our mediation analysis revealed that nephrotoxic medication use played a modest but significant role in mediating the relationship between HIV and CKD. These agents have well-documented nephrotoxic effects that could exacerbate kidney damage. Thus, the mediating role of nephrotoxic medications in the HIV – CKD pathway is likely driven by their nephrotoxicity. Importantly, the interaction between HIV and nephrotoxic medication use was statistically significant, suggesting that nephrotoxic drug exposure amplifies CKD risk in PLWH. These findings highlight the need for clinicians to routinely assess kidney function when prescribing or managing nephrotoxic medications in PLWH and to weigh renal risks alongside clinical benefits as part of personalized HIV care.

Following this key finding, we examined the broader relationships between HIV serostatus, depression, nephrotoxic medication use, and CKD. First, consistent with prior research [1], we observed a high prevalence of CKD, and a significantly higher prevalence of CKD in PLWH (18.1%) compared to PLWoH (9.7%). This disparity remained significant after adjusting for demographic, clinical, and behavioral covariates. Furthermore, the longitudinal analysis using GEE confirmed that HIV serostatus was a strong predictor of kidney function decline. Our findings are congruent with prior studies documenting that kidney disease in PLWH is a common and serious complication, with reports documenting kidney disease as the fourth leading cause of death in patients with AIDS prior to the use of effective ART and among individuals not using ART. Although HIV-associated nephropathy (HIVAN), especially in individuals carrying APOL1 risk alleles, was once the most common form of kidney injury in PLWH, its incidence has declined dramatically in the current ART era [23]. Today, other forms of CKD, such as diabetic kidney disease and hypertensive nephrosclerosis, are more frequently observed [14,23–27]. HIV can also lead to renal injury through mechanisms such as HIV-associated thrombotic microangiopathy (TMA), acute interstitial nephritis, immune complex-mediated glomerular disease, and sepsis-related acute tubular necrosis (ATN) due to immunosuppression [28].

Fortunately, compared to people without HIV, kidney dysfunction in PLWH has declined with the use of modern ART and may now progress at a similar or slower rate compared to those without HIV. However, many medications used to treat HIV and the complications of HIV can result in renal injury. Protease inhibitors, such as atazanavir, have been associated with crystal-induced obstructive acute kidney injury (AKI) [29]. Tenofovir (a nucleoside reverse transcriptase inhibitor) use has been implicated in tubulopathies causing Fanconi syndrome and nephrogenic diabetes insipidus [30,31]. Moreover, the presence of traditional risk factors such as hypertension, diabetes, and older age, remained critical determinants of CKD progression across both groups in our study.

Of note, individuals in our cohort with depression had a slightly lower prevalence of CKD compared to those without depression among PLWH. However, among PLWoH, CKD prevalence was also lower in those with depression compared to those without. These findings contrast with prior literature that suggests a strong link between depression and CKD progression [32–35], that is, there is a higher prevalence of depression as CKD worsens. One possible explanation for this discrepancy is that the MWCCS cohort includes individuals with more stable access to care and effective management of both HIV and mental health conditions. This may have attenuated the impact of depression on CKD progression. Studies such as those by Capuron et al. and Lustman et al. have suggested that while somatic depressive symptoms (e.g., fatigue, appetite loss) are more prevalent in conditions like diabetes, cognitive symptoms (e.g., sadness, hopelessness) may be more prominent in populations of persons with HIV [12,36]. In contrast, another group showed that in persons with cancer, the most optimal diagnostic tool for the identification of depression contained *both* somatic and cognitive symptoms (late insomnia, agitation, psychic anxiety, diurnal mood variation, depressed mood, and decreased libido) of the Hamilton Depression Rating Scale [37]. However, the lack of a significant mediating effect of depression on CKD progression in our analysis suggests that, although depression is highly prevalent among individuals with CKD, it may not independently contribute to disease progression [38,39]. One possible explanation is that participants with elevated depressive symptoms may have benefited from better access to supportive services or clinical follow-up that attenuated CKD risk. Nevertheless, because depression in this study was defined solely based on CES-D symptom scores rather than clinical diagnosis or treatment history, we cannot determine whether participants were receiving appropriate or effective depression management. The potential modifying effect of depression on CKD risk, particularly among individuals with HIV, remains unclear and warrants further investigation in future studies that incorporate both symptom-based and treatment-based assessments. In addition, some agents used in the management of depression or comorbid conditions may have nephrotoxic potential, which complicates causal interpretation and may introduce confounding by indication (e.g., medication use reflecting underlying comorbidity severity).

Despite the strong association between HIV and CKD, the mediation analysis indicated that nephrotoxic medication use accounted for a small but significant proportion of this relationship. These findings align with prior studies suggesting that certain nephrotoxic drugs, including commonly used antiretrovirals and other agents, may contribute to kidney injury over time [34,40–43].

### Strengths and limitations

Our study has several strengths. A key strength is our use of mediation analysis to disentangle the direct and indirect effects of HIV on CKD, thereby allowing us to gain new insights into the role of nephrotoxic medication exposure in this association among PLWH. Additionally, the longitudinal design allowed us to assess CKD progression over time and explore differences in HIV serostatus, depression, and medication use. The MWCCS cohort also represents a deeply phenotyped, diverse population.

However, there are limitations to consider. Our analytic sample required non-missing eGFR and key exposure/mediator measures, which may introduce selection bias if missingness relates to both HIV status and CKD risk. The observational nature of our study precludes causal inferences, and unmeasured confounding variables, such as levels of inflammation markers (e.g., C-reactive protein) and alternative kidney function measures (e.g., cystatin C), were not available in our dataset. Inflammation, medication adherence, and healthcare access may influence both depression and CKD risk. These markers may have provided a more accurate estimation of kidney function, particularly in individuals with low muscle mass [44]. Medication data were limited to nephrotoxic drug exposures captured in the MWCCS cohort. As a result, while we were able to quantify the mediating effect of nephrotoxic medications overall, we could not evaluate the impact of non-nephrotoxic psychiatric medications because they were not captured in the coded data during the study window. This restricts the generalizability of our findings and should be interpreted accordingly.

## Conclusion

In conclusion, our study reaffirms the strong association between HIV and CKD, even among PLWH successfully treated with ART, with most of the risk explained by direct effects rather than mediation through depression. While depression was highly prevalent among persons with CKD, it was not independently associated with CKD. In contrast, nephrotoxic medication use played a modest but significant mediating role, suggesting that the contribution of medications to CKD risk in PLWH is largely driven by their renal toxicity. These findings highlight the need for careful renal monitoring in patients prescribed medications with nephrotoxic potential. Future research should further explore the biological mechanisms linking HIV, depression, and kidney disease, with particular attention to both medication-related toxicity and systemic inflammatory pathways.

### Open science transparency statements

1. **Study Registration**

This study is a secondary data analysis of the Multicenter AIDS Cohort Study/ Women’s Interagency HIV Study Combined Cohort Study (MWCCS), a publicly funded, ongoing prospective cohort supported by the National Institutes of Health (U01-HL146193 and companion U01 sites). The MWCCS is registered at https://mwccs.org. No separate preregistration of this secondary analysis was required or conducted.

1. **Analytic Plan Registration**

The analytic plan, including model specifications for the generalized estimating equation (GEE) and counterfactual-based mediation analyses, was developed and approved internally by the MWCCS Data Analysis and Coordination Center and documented within the study’s data analysis request protocol. No formal preregistration was required or completed on an external registry (e.g., OSF or clinicaltrials.gov).

1. **Availability of Analytic Code**

Analysis was conducted using **SAS 9.4** and **R (version 4.3)**. The analytic code implementing the GEE and mediation analyses is available upon reasonable request from the corresponding author (Dr. Yue Pan, panyue@med.miami.edu) and will also be deposited in a public repository (e.g., Zenodo or OSF) upon publication.

1. **Availability of Materials**

All survey instruments, including the Center for Epidemiologic Studies Depression (CES-D) scale, and variable definitions (e.g., eGFR, nephrotoxic medication classification) are publicly documented within MWCCS data manuals and supplementary materials. These materials can be accessed through the MWCCS website or requested from the Data Analysis and Coordination Center.

## Supporting information

S1 Table Class-specific associations between nephrotoxic medication exposure and CKD (GEE Poisson models, N = 6,866 person-visits; 6,256 with complete covariate data). (DOCX)

S2 Table Classification of Medications by Nephrotoxic Properties, and Subcategories of Nephrotoxic Drugs. (DOCX)
