# Supplementary material for: HIV, nephrotoxic medications, and chronic kidney disease: Prevalence, risk factors, and mediation analyses among people with and without HIV enrolled in the Multicenter AIDS Cohort Study (MACS)/ Women’s Interagency HIV Study (WIHS) combined cohort study
Source: PLoS One. 2026 Jun 10;21(6):e0336467. doi: 10.1371/journal.pone.0336467 (PMC13252835; doi:10.1371/journal.pone.0336467)
Supplement: S1 Table — (DOCX) [file pone.0336467.s001.docx]

**Supplementary Table S1.** Class-specific associations between nephrotoxic medication exposure and CKD (GEE Poisson models, N = 6,866 person-visits; 6,256 with complete covariate data).

| **Drug Class** | **N Exposed** | **% Exposed** | **Events (Exposed)** | **Rate Ratio** | **95% CI Lower** | **95% CI Upper** | **P-value** |
| --- | --- | --- | --- | --- | --- | --- | --- |
| NSAIDs and Analgesics | 1,136 | 16.55% | 206 | 0.98 | 0.86 | 1.11 | 0.7338 |
| **ACE Inhibitors and ARBs** | **535** | **7.79%** | **127** | **1.32** | **1.12** | **1.56** | **0.0008** |
| **Diuretics** | **498** | **7.25%** | **126** | **1.49** | **1.25** | **1.77** | **<0.0001** |
| Nephrotoxic Antiretrovirals | 149 | 2.17% | 11 | 1.44 | 0.91 | 2.27 | 0.1227 |
| Nephrotoxic Antimicrobials | 334 | 4.86% | 46 | 0.87 | 0.64 | 1.18 | 0.3569 |
| **Other Nephrotoxic Medications** | **1,014** | **14.77%** | **171** | **0.86** | **0.75** | **1.00** | **0.0437** |

*Note: Models adjusted for HIV serostatus and age at visit. N exposed and events are based on the full analytic sample (visits 101–103) with non-missing eGFR.*

*† The 'Other Nephrotoxic Medications' category is heterogeneous and includes metformin, lithium, gabapentin, proton pump inhibitors, and others; the inverse association likely reflects confounding by indication.*

*‡ Nephrotoxic antiretrovirals result should be interpreted with caution given only 11 CKD events among 149 exposed person-visits.*
