# Supplementary material for: HIV, nephrotoxic medications, and chronic kidney disease: Prevalence, risk factors, and mediation analyses among people with and without HIV enrolled in the Multicenter AIDS Cohort Study (MACS)/ Women’s Interagency HIV Study (WIHS) combined cohort study
Source: PLoS One. 2026 Jun 10;21(6):e0336467. doi: 10.1371/journal.pone.0336467 (PMC13252835; doi:10.1371/journal.pone.0336467)
Supplement: S2 Table — (DOCX) [file pone.0336467.s002.docx]

**Supplementary Table 2: Classification of Medications by Nephrotoxic Properties, and Subcategories of Nephrotoxic Drugs**

| Nephrotoxicity_Drug | Nephro_Subcategory_Label | nephro_nsaid | nephro_acei_arb | nephro_diuretic | nephro_arv | nephro_antimicrobial | nephro_other | nephro_any_subcat |
| --- | --- | --- | --- | --- | --- | --- | --- | --- |
| ASA | NSAIDs / analgesics | 1 | 0 | 0 | 0 | 0 | 0 | 1 |
| ACETAZOLAMID | Diuretics (incl. acetazolamide/spironolactone) | 0 | 0 | 1 | 0 | 0 | 0 | 1 |
| ACYCLOVIR | Antimicrobials (ABX/antivirals/other) | 0 | 0 | 0 | 0 | 1 | 0 | 1 |
| ADVIL | NSAIDs / analgesics | 1 | 0 | 0 | 0 | 0 | 0 | 1 |
| ALEVE | NSAIDs / analgesics | 1 | 0 | 0 | 0 | 0 | 0 | 1 |
| ALEVE-D | NSAIDs / analgesics | 1 | 0 | 0 | 0 | 0 | 0 | 1 |
| ALLOPURINOL | Other nephrotoxic meds (metformin, PPIs, lithium, etc.) | 0 | 0 | 0 | 0 | 0 | 1 | 1 |
| ALOPURINOL | Other nephrotoxic meds (metformin, PPIs, lithium, etc.) | 0 | 0 | 0 | 0 | 0 | 1 | 1 |
| AMOX | Antimicrobials (ABX/antivirals/other) | 0 | 0 | 0 | 0 | 1 | 0 | 1 |
| ANACIN | NSAIDs / analgesics | 1 | 0 | 0 | 0 | 0 | 0 | 1 |
| ASPIRIN | NSAIDs / analgesics | 1 | 0 | 0 | 0 | 0 | 0 | 1 |
| AZOR | ACE inhibitors / ARBs | 0 | 1 | 0 | 0 | 0 | 0 | 1 |
| BACTRIM | Antimicrobials (ABX/antivirals/other) | 0 | 0 | 0 | 0 | 1 | 0 | 1 |
| BAYER ASPIRIN | NSAIDs / analgesics | 1 | 0 | 0 | 0 | 0 | 0 | 1 |
| BAYER BACK & BODY | NSAIDs / analgesics | 1 | 0 | 0 | 0 | 0 | 0 | 1 |
| BENICAR | ACE inhibitors / ARBs | 0 | 1 | 0 | 0 | 0 | 0 | 1 |
| BIKTARVY | Nephrotoxic antiretrovirals (tenofovir-based/other) | 0 | 0 | 0 | 1 | 0 | 0 | 1 |
| BISOPROLOL-HYDROCHLOROTHIAZIDE | Diuretics (incl. acetazolamide/spironolactone) | 0 | 0 | 1 | 0 | 0 | 0 | 1 |
| CELEBREX | NSAIDs / analgesics | 1 | 0 | 0 | 0 | 0 | 0 | 1 |
| CIPRO | Antimicrobials (ABX/antivirals/other) | 0 | 0 | 0 | 0 | 1 | 0 | 1 |
| CIPROFLOXACINE | Antimicrobials (ABX/antivirals/other) | 0 | 0 | 0 | 0 | 1 | 0 | 1 |
| COMPLERA | Nephrotoxic antiretrovirals (tenofovir-based/other) | 0 | 0 | 0 | 1 | 0 | 0 | 1 |
| DESCOVY | Nephrotoxic antiretrovirals (tenofovir-based/other) | 0 | 0 | 0 | 1 | 0 | 0 | 1 |
| DICLOPHENAC | NSAIDs / analgesics | 1 | 0 | 0 | 0 | 0 | 0 | 1 |
| ECOTRIN | NSAIDs / analgesics | 1 | 0 | 0 | 0 | 0 | 0 | 1 |
| ENTRESTO | ACE inhibitors / ARBs | 0 | 1 | 0 | 0 | 0 | 0 | 1 |
| ENVARSUS | Other nephrotoxic meds (metformin, PPIs, lithium, etc.) | 0 | 0 | 0 | 0 | 0 | 1 | 1 |
| EXCEDRIN | NSAIDs / analgesics | 1 | 0 | 0 | 0 | 0 | 0 | 1 |
| EXFORGE HCT | ACE inhibitors / ARBs | 0 | 1 | 0 | 0 | 0 | 0 | 1 |
| FARXIGA | Other nephrotoxic meds (metformin, PPIs, lithium, etc.) | 0 | 0 | 0 | 0 | 0 | 1 | 1 |
| FUROSEMID | Diuretics (incl. acetazolamide/spironolactone) | 0 | 0 | 1 | 0 | 0 | 0 | 1 |
| FUROSEMIDE | Diuretics (incl. acetazolamide/spironolactone) | 0 | 0 | 1 | 0 | 0 | 0 | 1 |
| GABAPENTINE | Other nephrotoxic meds (metformin, PPIs, lithium, etc.) | 0 | 0 | 0 | 0 | 0 | 1 | 1 |
| GENVOYA | Nephrotoxic antiretrovirals (tenofovir-based/other) | 0 | 0 | 0 | 1 | 0 | 0 | 1 |
| HCTZ | Diuretics (incl. acetazolamide/spironolactone) | 0 | 0 | 1 | 0 | 0 | 0 | 1 |
| HCTZ TRIAMTERENE | Diuretics (incl. acetazolamide/spironolactone) | 0 | 0 | 1 | 0 | 0 | 0 | 1 |
| HYDROCHLOROTHIAZIDE/LISINOPRIL | ACE inhibitors / ARBs | 0 | 1 | 0 | 0 | 0 | 0 | 1 |
| HYDROCHLOROTHIAZIDE | Diuretics (incl. acetazolamide/spironolactone) | 0 | 0 | 1 | 0 | 0 | 0 | 1 |
| HYDROCHLOROCHIN | Antimicrobials (ABX/antivirals/other) | 0 | 0 | 0 | 0 | 1 | 0 | 1 |
| HYZAAR | ACE inhibitors / ARBs | 0 | 1 | 0 | 0 | 0 | 0 | 1 |
| IBUPROFEN | NSAIDs / analgesics | 1 | 0 | 0 | 0 | 0 | 0 | 1 |
| IBUPROPHEN | NSAIDs / analgesics | 1 | 0 | 0 | 0 | 0 | 0 | 1 |
| LASIX | Diuretics (incl. acetazolamide/spironolactone) | 0 | 0 | 1 | 0 | 0 | 0 | 1 |
| LISINOPRIL | ACE inhibitors / ARBs | 0 | 1 | 0 | 0 | 0 | 0 | 1 |
| LOSARTAN | ACE inhibitors / ARBs | 0 | 1 | 0 | 0 | 0 | 0 | 1 |
| METFORMIN | Other nephrotoxic meds (metformin, PPIs, lithium, etc.) | 0 | 0 | 0 | 0 | 0 | 1 | 1 |
| METFORMIN | Other nephrotoxic meds (metformin, PPIs, lithium, etc.) | 0 | 0 | 0 | 0 | 0 | 1 | 1 |
| METFORMINE | Other nephrotoxic meds (metformin, PPIs, lithium, etc.) | 0 | 0 | 0 | 0 | 0 | 1 | 1 |
| MOTRIN | NSAIDs / analgesics | 1 | 0 | 0 | 0 | 0 | 0 | 1 |
| NAPROSIN | NSAIDs / analgesics | 1 | 0 | 0 | 0 | 0 | 0 | 1 |
| NAPROSYN | NSAIDs / analgesics | 1 | 0 | 0 | 0 | 0 | 0 | 1 |
| NAPROXEN | NSAIDs / analgesics | 1 | 0 | 0 | 0 | 0 | 0 | 1 |
| NEURONTIN | Other nephrotoxic meds (metformin, PPIs, lithium, etc.) | 0 | 0 | 0 | 0 | 0 | 1 | 1 |
| NEXPLANON | Other nephrotoxic meds (metformin, PPIs, lithium, etc.) | 0 | 0 | 0 | 0 | 0 | 1 | 1 |
| OMEPRAZOLE | Other nephrotoxic meds (metformin, PPIs, lithium, etc.) | 0 | 0 | 0 | 0 | 0 | 1 | 1 |
| OMEPRAZOL | Other nephrotoxic meds (metformin, PPIs, lithium, etc.) | 0 | 0 | 0 | 0 | 0 | 1 | 1 |
| OMEPRAZOLE | Other nephrotoxic meds (metformin, PPIs, lithium, etc.) | 0 | 0 | 0 | 0 | 0 | 1 | 1 |
| PREVACID | Other nephrotoxic meds (metformin, PPIs, lithium, etc.) | 0 | 0 | 0 | 0 | 0 | 1 | 1 |
| PRILOSEC | Other nephrotoxic meds (metformin, PPIs, lithium, etc.) | 0 | 0 | 0 | 0 | 0 | 1 | 1 |
| PRINIVIL | ACE inhibitors / ARBs | 0 | 1 | 0 | 0 | 0 | 0 | 1 |
| PRINZIDE | ACE inhibitors / ARBs | 0 | 1 | 0 | 0 | 0 | 0 | 1 |
| PROTONIX | Other nephrotoxic meds (metformin, PPIs, lithium, etc.) | 0 | 0 | 0 | 0 | 0 | 1 | 1 |
| RECLAST | Other nephrotoxic meds (metformin, PPIs, lithium, etc.) | 0 | 0 | 0 | 0 | 0 | 1 | 1 |
| REGLAN | Other nephrotoxic meds (metformin, PPIs, lithium, etc.) | 0 | 0 | 0 | 0 | 0 | 1 | 1 |
| REMDESIVIR | Antimicrobials (ABX/antivirals/other) | 0 | 0 | 0 | 0 | 1 | 0 | 1 |
| SPIRONOLACTONE | Diuretics (incl. acetazolamide/spironolactone) | 0 | 0 | 1 | 0 | 0 | 0 | 1 |
| SULFAMETHOXAZOLE/TRIMETHOPRIM | Antimicrobials (ABX/antivirals/other) | 0 | 0 | 0 | 0 | 1 | 0 | 1 |
| SPIROLACTONE | Diuretics (incl. acetazolamide/spironolactone) | 0 | 0 | 1 | 0 | 0 | 0 | 1 |
| SULFAMETHOXAZOLE | Antimicrobials (ABX/antivirals/other) | 0 | 0 | 0 | 0 | 1 | 0 | 1 |
| SYMTUZA | Nephrotoxic antiretrovirals (tenofovir-based/other) | 0 | 0 | 0 | 1 | 0 | 0 | 1 |
| TIVICAY | Nephrotoxic antiretrovirals (tenofovir-based/other) | 0 | 0 | 0 | 1 | 0 | 0 | 1 |
| TRIBENZOR | ACE inhibitors / ARBs | 0 | 1 | 0 | 0 | 0 | 0 | 1 |
| TRIUMEQ | Nephrotoxic antiretrovirals (tenofovir-based/other) | 0 | 0 | 0 | 1 | 0 | 0 | 1 |
| TRUVADA | Nephrotoxic antiretrovirals (tenofovir-based/other) | 0 | 0 | 0 | 1 | 0 | 0 | 1 |
| VALTREX | Antimicrobials (ABX/antivirals/other) | 0 | 0 | 0 | 0 | 1 | 0 | 1 |
| VEMLIDY | Nephrotoxic antiretrovirals (tenofovir-based/other) | 0 | 0 | 0 | 1 | 0 | 0 | 1 |
| VIMPAT | Other nephrotoxic meds (metformin, PPIs, lithium, etc.) | 0 | 0 | 0 | 0 | 0 | 1 | 1 |
| VOLTAREN | NSAIDs / analgesics | 1 | 0 | 0 | 0 | 0 | 0 | 1 |
| ZOVIRAX | Antimicrobials (ABX/antivirals/other) | 0 | 0 | 0 | 0 | 1 | 0 | 1 |
| ACETAZOLAMIDE | Diuretics (incl. acetazolamide/spironolactone) | 0 | 0 | 1 | 0 | 0 | 0 | 1 |
| ACETAZOLAMIDE | Diuretics (incl. acetazolamide/spironolactone) | 0 | 0 | 1 | 0 | 0 | 0 | 1 |
| ACYCLOVIR | Antimicrobials (ABX/antivirals/other) | 0 | 0 | 0 | 0 | 1 | 0 | 1 |
| ACYCLOVIR 400 MG | Antimicrobials (ABX/antivirals/other) | 0 | 0 | 0 | 0 | 1 | 0 | 1 |
| ADVIL | NSAIDs / analgesics | 1 | 0 | 0 | 0 | 0 | 0 | 1 |
| ALEVE | NSAIDs / analgesics | 1 | 0 | 0 | 0 | 0 | 0 | 1 |
| ALLOPURINOL | Other nephrotoxic meds (metformin, PPIs, lithium, etc.) | 0 | 0 | 0 | 0 | 0 | 1 | 1 |
| AMANTADINE | Other nephrotoxic meds (metformin, PPIs, lithium, etc.) | 0 | 0 | 0 | 0 | 0 | 1 | 1 |
| AMOXICILLIN/CLAVULANATE | Antimicrobials (ABX/antivirals/other) | 0 | 0 | 0 | 0 | 1 | 0 | 1 |
| ASA | NSAIDs / analgesics | 1 | 0 | 0 | 0 | 0 | 0 | 1 |
| ASPIRIN | NSAIDs / analgesics | 1 | 0 | 0 | 0 | 0 | 0 | 1 |
| ASPIRIN 81 MG | NSAIDs / analgesics | 1 | 0 | 0 | 0 | 0 | 0 | 1 |
| ASPIRIN 81 MG | NSAIDs / analgesics | 1 | 0 | 0 | 0 | 0 | 0 | 1 |
| BACTRIM | Antimicrobials (ABX/antivirals/other) | 0 | 0 | 0 | 0 | 1 | 0 | 1 |
| BAYER ASPIRIN | NSAIDs / analgesics | 1 | 0 | 0 | 0 | 0 | 0 | 1 |
| BENAZAPRIL | ACE inhibitors / ARBs | 0 | 1 | 0 | 0 | 0 | 0 | 1 |
| BIKTARVY | Nephrotoxic antiretrovirals (tenofovir-based/other) | 0 | 0 | 0 | 1 | 0 | 0 | 1 |
| BISOPROLOL/HYDROCHLOROTHIAZIDE | Diuretics (incl. acetazolamide/spironolactone) | 0 | 0 | 1 | 0 | 0 | 0 | 1 |
| CELEBREX | NSAIDs / analgesics | 1 | 0 | 0 | 0 | 0 | 0 | 1 |
| CELECOXIB | NSAIDs / analgesics | 1 | 0 | 0 | 0 | 0 | 0 | 1 |
| DESCOVY | Nephrotoxic antiretrovirals (tenofovir-based/other) | 0 | 0 | 0 | 1 | 0 | 0 | 1 |
| DICROFENAC | NSAIDs / analgesics | 1 | 0 | 0 | 0 | 0 | 0 | 1 |
| DIGOXIN | Other nephrotoxic meds (metformin, PPIs, lithium, etc.) | 0 | 0 | 0 | 0 | 0 | 1 | 1 |
| DIHYDROCHLOROTHIAZIDE | Diuretics (incl. acetazolamide/spironolactone) | 0 | 0 | 1 | 0 | 0 | 0 | 1 |
| ENALAPRIL | ACE inhibitors / ARBs | 0 | 1 | 0 | 0 | 0 | 0 | 1 |
| ENTRESTO | ACE inhibitors / ARBs | 0 | 1 | 0 | 0 | 0 | 0 | 1 |
| EXCEDRIN | NSAIDs / analgesics | 1 | 0 | 0 | 0 | 0 | 0 | 1 |
| FARXIGA | Other nephrotoxic meds (metformin, PPIs, lithium, etc.) | 0 | 0 | 0 | 0 | 0 | 1 | 1 |
| FOSINOPRIL/HYDROCHLOROTHIAZIDE | ACE inhibitors / ARBs | 0 | 1 | 0 | 0 | 0 | 0 | 1 |
| FUROSEMIDE | Diuretics (incl. acetazolamide/spironolactone) | 0 | 0 | 1 | 0 | 0 | 0 | 1 |
| GABAPENTIN | Other nephrotoxic meds (metformin, PPIs, lithium, etc.) | 0 | 0 | 0 | 0 | 0 | 1 | 1 |
| GENVOYA | Nephrotoxic antiretrovirals (tenofovir-based/other) | 0 | 0 | 0 | 1 | 0 | 0 | 1 |
| HCTZ | Diuretics (incl. acetazolamide/spironolactone) | 0 | 0 | 1 | 0 | 0 | 0 | 1 |
| HYDROCHLOROTHIAZIDE | Diuretics (incl. acetazolamide/spironolactone) | 0 | 0 | 1 | 0 | 0 | 0 | 1 |
| HYDROCHLOROTHIAZIDE/TRIAMTERENE | Diuretics (incl. acetazolamide/spironolactone) | 0 | 0 | 1 | 0 | 0 | 0 | 1 |
| HYDROCHLOROTHIAZIDE | Diuretics (incl. acetazolamide/spironolactone) | 0 | 0 | 1 | 0 | 0 | 0 | 1 |
| HYDROXYCHLOROQUINE | Antimicrobials (ABX/antivirals/other) | 0 | 0 | 0 | 0 | 1 | 0 | 1 |
| HYZAAR | ACE inhibitors / ARBs | 0 | 1 | 0 | 0 | 0 | 0 | 1 |
| IBUPROFEN | NSAIDs / analgesics | 1 | 0 | 0 | 0 | 0 | 0 | 1 |
| IBUPROFEN 800 MG | NSAIDs / analgesics | 1 | 0 | 0 | 0 | 0 | 0 | 1 |
| IBUPROPHEN | NSAIDs / analgesics | 1 | 0 | 0 | 0 | 0 | 0 | 1 |
| INDOMETHACIN | NSAIDs / analgesics | 1 | 0 | 0 | 0 | 0 | 0 | 1 |
| KETOROLAC | NSAIDs / analgesics | 1 | 0 | 0 | 0 | 0 | 0 | 1 |
| KETOROLAC TROMETHAMINE | NSAIDs / analgesics | 1 | 0 | 0 | 0 | 0 | 0 | 1 |
| LANSOPRAZOL | Other nephrotoxic meds (metformin, PPIs, lithium, etc.) | 0 | 0 | 0 | 0 | 0 | 1 | 1 |
| LASIX | Diuretics (incl. acetazolamide/spironolactone) | 0 | 0 | 1 | 0 | 0 | 0 | 1 |
| LISINOPRIL | ACE inhibitors / ARBs | 0 | 1 | 0 | 0 | 0 | 0 | 1 |
| LITHIUM | Other nephrotoxic meds (metformin, PPIs, lithium, etc.) | 0 | 0 | 0 | 0 | 0 | 1 | 1 |
| LITHIUM CARBONATE | Other nephrotoxic meds (metformin, PPIs, lithium, etc.) | 0 | 0 | 0 | 0 | 0 | 1 | 1 |
| LOSARTAN | ACE inhibitors / ARBs | 0 | 1 | 0 | 0 | 0 | 0 | 1 |
| LOSARTAN POTASSIUM | ACE inhibitors / ARBs | 0 | 1 | 0 | 0 | 0 | 0 | 1 |
| METFORMIN | Other nephrotoxic meds (metformin, PPIs, lithium, etc.) | 0 | 0 | 0 | 0 | 0 | 1 | 1 |
| METFORMIN | Other nephrotoxic meds (metformin, PPIs, lithium, etc.) | 0 | 0 | 0 | 0 | 0 | 1 | 1 |
| METFORMIN HYDROCHLORIDE | Other nephrotoxic meds (metformin, PPIs, lithium, etc.) | 0 | 0 | 0 | 0 | 0 | 1 | 1 |
| METFORMINE | Other nephrotoxic meds (metformin, PPIs, lithium, etc.) | 0 | 0 | 0 | 0 | 0 | 1 | 1 |
| METHOTREXATE | Other nephrotoxic meds (metformin, PPIs, lithium, etc.) | 0 | 0 | 0 | 0 | 0 | 1 | 1 |
| MOBIC | NSAIDs / analgesics | 1 | 0 | 0 | 0 | 0 | 0 | 1 |
| MOTRIN | NSAIDs / analgesics | 1 | 0 | 0 | 0 | 0 | 0 | 1 |
| NAPROSIN | NSAIDs / analgesics | 1 | 0 | 0 | 0 | 0 | 0 | 1 |
| NAPROXEN | NSAIDs / analgesics | 1 | 0 | 0 | 0 | 0 | 0 | 1 |
| NEOMYCIN | Antimicrobials (ABX/antivirals/other) | 0 | 0 | 0 | 0 | 1 | 0 | 1 |
| NEURONTIN | Other nephrotoxic meds (metformin, PPIs, lithium, etc.) | 0 | 0 | 0 | 0 | 0 | 1 | 1 |
| NEXPLANON | Other nephrotoxic meds (metformin, PPIs, lithium, etc.) | 0 | 0 | 0 | 0 | 0 | 1 | 1 |
| NITROFURANTOIN | Antimicrobials (ABX/antivirals/other) | 0 | 0 | 0 | 0 | 1 | 0 | 1 |
| ODEFSEY | Nephrotoxic antiretrovirals (tenofovir-based/other) | 0 | 0 | 0 | 1 | 0 | 0 | 1 |
| OFLOXACIN | NSAIDs / analgesics; Antimicrobials (ABX/antivirals/other) | 1 | 0 | 0 | 0 | 1 | 0 | 1 |
| OLMESARTAN | ACE inhibitors / ARBs | 0 | 1 | 0 | 0 | 0 | 0 | 1 |
| OMEPRAZOLE | Other nephrotoxic meds (metformin, PPIs, lithium, etc.) | 0 | 0 | 0 | 0 | 0 | 1 | 1 |
| OMESARTAN | ACE inhibitors / ARBs | 0 | 1 | 0 | 0 | 0 | 0 | 1 |
| PANTOPRAZOLE | Other nephrotoxic meds (metformin, PPIs, lithium, etc.) | 0 | 0 | 0 | 0 | 0 | 1 | 1 |
| PREVACID | Other nephrotoxic meds (metformin, PPIs, lithium, etc.) | 0 | 0 | 0 | 0 | 0 | 1 | 1 |
| PRILOSEC | Other nephrotoxic meds (metformin, PPIs, lithium, etc.) | 0 | 0 | 0 | 0 | 0 | 1 | 1 |
| PRINIVIL | ACE inhibitors / ARBs | 0 | 1 | 0 | 0 | 0 | 0 | 1 |
| PROTONIX | Other nephrotoxic meds (metformin, PPIs, lithium, etc.) | 0 | 0 | 0 | 0 | 0 | 1 | 1 |
| QUINAPRIL | ACE inhibitors / ARBs | 0 | 1 | 0 | 0 | 0 | 0 | 1 |
| RAMIPRIL | ACE inhibitors / ARBs | 0 | 1 | 0 | 0 | 0 | 0 | 1 |
| RELAFEN | NSAIDs / analgesics | 1 | 0 | 0 | 0 | 0 | 0 | 1 |
| REMDESIVIR | Antimicrobials (ABX/antivirals/other) | 0 | 0 | 0 | 0 | 1 | 0 | 1 |
| RIFAMPIN | Antimicrobials (ABX/antivirals/other) | 0 | 0 | 0 | 0 | 1 | 0 | 1 |
| SOTALOL | Diuretics (incl. acetazolamide/spironolactone) | 0 | 0 | 1 | 0 | 0 | 0 | 1 |
| SPIROLACTONE | Diuretics (incl. acetazolamide/spironolactone) | 0 | 0 | 1 | 0 | 0 | 0 | 1 |
| SPIRONOLACTONE | Diuretics (incl. acetazolamide/spironolactone) | 0 | 0 | 1 | 0 | 0 | 0 | 1 |
| SPIRULINA | Other nephrotoxic meds (metformin, PPIs, lithium, etc.) | 0 | 0 | 0 | 0 | 0 | 1 | 1 |
| SULFAMETHOXAZOLE | Antimicrobials (ABX/antivirals/other) | 0 | 0 | 0 | 0 | 1 | 0 | 1 |
| SULFAMETHOXAZOLE-TRIMETHOPRIM | Antimicrobials (ABX/antivirals/other) | 0 | 0 | 0 | 0 | 1 | 0 | 1 |
| SULFAMETHOXAZOLE/TRIMETHOPRIM | Antimicrobials (ABX/antivirals/other) | 0 | 0 | 0 | 0 | 1 | 0 | 1 |
| SULFASALAZINE | Antimicrobials (ABX/antivirals/other) | 0 | 0 | 0 | 0 | 1 | 0 | 1 |
| SULINDAC | NSAIDs / analgesics | 1 | 0 | 0 | 0 | 0 | 0 | 1 |
| TENOFOVIR | Nephrotoxic antiretrovirals (tenofovir-based/other) | 0 | 0 | 0 | 1 | 0 | 0 | 1 |
| TIVICAY | Nephrotoxic antiretrovirals (tenofovir-based/other) | 0 | 0 | 0 | 1 | 0 | 0 | 1 |
| TRIBENZOR | ACE inhibitors / ARBs | 0 | 1 | 0 | 0 | 0 | 0 | 1 |
| TRUVADA | Nephrotoxic antiretrovirals (tenofovir-based/other) | 0 | 0 | 0 | 1 | 0 | 0 | 1 |
| VALACYCLOVIR | Antimicrobials (ABX/antivirals/other) | 0 | 0 | 0 | 0 | 1 | 0 | 1 |
| VALACICLOVIR | Antimicrobials (ABX/antivirals/other) | 0 | 0 | 0 | 0 | 1 | 0 | 1 |
| VALACYCLOVIR | Antimicrobials (ABX/antivirals/other) | 0 | 0 | 0 | 0 | 1 | 0 | 1 |
| VALACYCLOVIR 500 MG | Antimicrobials (ABX/antivirals/other) | 0 | 0 | 0 | 0 | 1 | 0 | 1 |
| VALACYCLOVIR HCL | Antimicrobials (ABX/antivirals/other) | 0 | 0 | 0 | 0 | 1 | 0 | 1 |
| VALSARTAN | ACE inhibitors / ARBs | 0 | 1 | 0 | 0 | 0 | 0 | 1 |
| VALTREX | Antimicrobials (ABX/antivirals/other) | 0 | 0 | 0 | 0 | 1 | 0 | 1 |
| VANCOMYCIN | Antimicrobials (ABX/antivirals/other) | 0 | 0 | 0 | 0 | 1 | 0 | 1 |
| VEMLIDY | Nephrotoxic antiretrovirals (tenofovir-based/other) | 0 | 0 | 0 | 1 | 0 | 0 | 1 |
| VIMPAT | Other nephrotoxic meds (metformin, PPIs, lithium, etc.) | 0 | 0 | 0 | 0 | 0 | 1 | 1 |
| VIREAD | Nephrotoxic antiretrovirals (tenofovir-based/other) | 0 | 0 | 0 | 1 | 0 | 0 | 1 |
| VOLTAREN | NSAIDs / analgesics | 1 | 0 | 0 | 0 | 0 | 0 | 1 |
| ZOVIRAX | Antimicrobials (ABX/antivirals/other) | 0 | 0 | 0 | 0 | 1 | 0 | 1 |

*

Nephrotoxicity_Drug = "Any Nephrotoxic Medication (Drug-Level Indicator)"

Nephro_Subcategory_Label = "Nephrotoxic Drug Subcategory (Text Classification)"

nephro_nsaid = "NSAIDs and Analgesics (Nephrotoxic Class Indicator)"

nephro_acei_arb = "ACE Inhibitors / ARBs (Nephrotoxic Class Indicator)"

nephro_diuretic = "Diuretics (Nephrotoxic Class Indicator)"

nephro_arv = "Nephrotoxic Antiretrovirals (Nephrotoxic Class Indicator)"

nephro_antimicrobial = "Antimicrobials and Anti-infective Agents (Nephrotoxic Class Indicator)"

nephro_other = "Other Nephrotoxic Medications (Nephrotoxic Class Indicator)"

nephro_any_subcat = "Any Nephrotoxic Subcategory Indicator";
