# Supplementary material for: HIV, nephrotoxic medications, and chronic kidney disease: Prevalence, risk factors, and mediation analyses among people with and without HIV enrolled in the Multicenter AIDS Cohort Study (MACS)/ Women’s Interagency HIV Study (WIHS) combined cohort study
Source: PLoS One. 2026 Jun 10;21(6):e0336467. doi: 10.1371/journal.pone.0336467 (PMC13252835; doi:10.1371/journal.pone.0336467)
Supplement: Attachment — Submitted filename: PONE-D-25-57826_Review.docx [file pone.0336467.s003.docx]

**General Comments**

This manuscript addresses an important and clinically relevant question regarding the relationships among HIV serostatus, depression, nephrotoxic medication use, and chronic kidney disease (CKD) within the MWCCS cohort. The topic is timely, and the dataset is rich. I commend the authors for this manuscript. However, several methodological and interpretive issues require clarification before the findings can be fully evaluated. Strengthening the transparency of the analytic approach and improving the organization of the manuscript would substantially enhance its clarity and scientific contribution.

**Major Comments**

**1. Clarification of GEE model specification and adjustment set**

The manuscript reports relative risks from GEE models but does not clearly specify whether these estimates are adjusted or unadjusted. Although several covariates (e.g., age, race, diabetes, income) are mentioned in the Results, the Methods section does not explicitly state which variables were included simultaneously in the GEE model. Because age is a dominant determinant of CKD and is strongly

correlated with many of the reported covariates, it is difficult to interpret the associations without knowing the exact adjustment set. Some of the observed associations may simply reflect age structure rather than independent effects.

I recommend that the authors clearly list all covariates included in each GEE model, distinguish unadjusted from adjusted estimates, and provide a rationale for the chosen adjustment set. Presenting both unadjusted and adjusted RRs in a table would greatly improve interpretability.

**2. Mediation analysis: Potential over‑adjustment and model transparency**

The mediation analysis requires clearer description. It is not evident whether depressive symptoms and nephrotoxic medication use were included as covariates in the primary GEE model. If mediators were adjusted for in the outcome model used to estimate the total effect, this would bias the estimated direct effect of HIV on CKD and undermine the validity of the mediation analysis. Standard counterfactual mediation approaches require that mediators not be controlled for in the total‑effect model.

The authors should explicitly state:

- Which covariates were included in the GEE model estimating the total effect
- Which covariates were included in the mediator and outcome models
- Whether any variables on the causal pathway were inadvertently adjusted for

Including a DAG or a brief justification of the adjustment set would strengthen the causal interpretation.

**3. Construction and heterogeneity of the nephrotoxic medication exposure**

The definition of nephrotoxic medication exposure requires more detail. It is unclear whether the exposure reflects any use, cumulative use, dose, duration, or specific drug classes. Nephrotoxic medications are heterogeneous, and collapsing them into a single binary indicator risks substantial exposure misclassification. This is particularly important because the mediation analysis treats this variable as a mechanistic pathway.

The authors should consider:

- Describing how the exposure was constructed
- Justifying the chosen categorization
- Considering sensitivity analyses using alternative definitions (e.g., cumulative burden, class‑specific indicators)
- Discussing the implications of heterogeneity within the exposure

**4. Interpretation of depression findings and internal inconsistency**

The discussion presents conflicting explanations for the null association between depression and CKD. On one hand, the authors suggest that effective depression treatment in the cohort may explain the lack of association. On the other hand, they acknowledge that depression was defined solely by CES‑D scores, without information on diagnosis or treatment, making it impossible to determine whether participants were receiving appropriate care.

These two statements are contradictory. Moreover, part of the hypothesized link between depression and CKD involves the use of nephrotoxic antidepressants, which complicates the interpretation of “effective treatment” as a protective factor.

The authors should consider reconciling these points and provide a more coherent interpretation of the null finding.

**5. Abstract should explicitly state the null finding for depression**

Given that depression is central to the study’s rationale, title, and keywords, the abstract should explicitly state that depressive symptoms were not associated with CKD in the adjusted analyses and did not mediate the HIV-CKD relationship. This omission may mislead readers and should be corrected.

**Moderate Comments**

**6. Flow chart of recruitment and analytic sample**

The description of recruitment, retention, and analytic sample construction is complex. A flow chart would greatly improve clarity by illustrating:

- the number of participants at each visit
- how many were new vs. returning
- how many were included in each analysis
- reasons for exclusion

This would help readers understand the analytic dataset.

**7. Organization of the Discussion**

The discussion would benefit from beginning with a concise summary of the study’s aims and key findings before moving into interpretation. This would help orient readers and improve flow.

Additionally, the section discussing the etiology of nephropathy in the pre‑ART era seems more appropriate for the introduction section, where it would better contextualize the biological plausibility of HIV‑related kidney injury.

**Minor Comments**

There is an incomplete sentence in the discussion:

“Studies such as those by Capuron et al. and Lustman et al. have suggested that while somatic depressive symptoms (e.g., fatigue, appetite loss) are more prevalent in conditions like diabetes, cognitive symptoms (e.g., sadness, hopelessness) may be more prominent in populations of persons with [12, 32].”

**Overall Assessment**

This manuscript addresses an important topic and has the potential to make a meaningful contribution. Clarifying the analytic methods, improving internal consistency, and strengthening the organization would substantially enhance the rigor and interpretability of the findings.
