# Supplementary material for: HIV, nephrotoxic medications, and chronic kidney disease: Prevalence, risk factors, and mediation analyses among people with and without HIV enrolled in the Multicenter AIDS Cohort Study (MACS)/ Women’s Interagency HIV Study (WIHS) combined cohort study
Source: PLoS One. 2026 Jun 10;21(6):e0336467. doi: 10.1371/journal.pone.0336467 (PMC13252835; doi:10.1371/journal.pone.0336467)
Supplement: Attachment — Submitted filename: Response to reviewer R2.docx [file pone.0336467.s005.docx]

**Response to Reviewers and Editor Comments**

**Reviewer #1:
1. It is not clear whether the current analysis was on one or on repeated measures. In the methods section, you note that visit 101 data was analysed, yet in the statistical modeling, results, and discussion, there are mentions of repeated measures.**

Response: We apologize for the confusion. The study utilizes two complementary analytic components. Baseline prevalence was assessed using Visit 101 data only. However, the Generalized Estimating Equations (GEE) models used longitudinal data from three consecutive visits (101, 102, and 103) to account for within-subject correlation. We have revised the Methods section to clearly distinguish between the baseline prevalence analysis and the longitudinal GEE modeling, and we explicitly state that the GEE analyses are conducted as separate univariate models (one predictor at a time).

**2. Please clarify why visit 101 was chosen as "baseline".**

Response: Visit 101 (November 16, 2020-September 30, 2021) was selected as the baseline because it represents the first MWCCS visit in the consolidated MACS/WIHS cohort with harmonized measures used in the current analysis, providing a consistent anchor for both baseline prevalence estimation and repeated-measures modeling.

**3. It would be important to clarify how the bidirectional relationships between CKD and depression, nephrotoxic medications and CKD, and hypertension and nephrotoxic medications were modeled.**

Response: We acknowledge the potential for bidirectionality and confounding by indication (e.g., CKD influencing medication choice and depressive symptoms; hypertension influencing nephrotoxic medication exposure). Our repeated-measures GEE models estimate contemporaneous associations at each visit and cannot fully establish temporal ordering; therefore, we interpret GEE findings as associative rather than causal. To improve transparency regarding causal assumptions for mediation, we added a prespecified directed acyclic graph (DAG; Figure 2) and explicitly state that the DAG is used to define the adjustment set for the counterfactual mediation analyses. Additionally, our causal mediation analysis used counterfactual-based approaches to estimate the pathways from HIV status to CKD through mediators while adjusting for baseline confounders. We strengthened the manuscript language to explicitly acknowledge reverse causation and confounding by indication, particularly for medication classes commonly prescribed for kidney-related conditions (e.g., ACEi/ARB, diuretics).

**4. It is not clear how nephrotoxic medications, hypertension, diabetes, etc were defined, and misclassification was minimized, or by how much did it affect the results?**

Response: We have provided specific definitions in the Methods: CKD was operationalized as eGFR <60 mL/min/1.73 m² at the study visit (CKD-EPI). Nephrotoxic Medications: Identified via a modified Delphi method and expert consensus. Hypertension/Diabetes: Based on medical diagnoses or medication use. We have added a "Limitations" section discussing how self-reported history for some covariates might lead to misclassification and how this could potentially bias results toward the null.

**5. What is the reliability and consistency of CES-D in the identification of depression in people with HIV?**

Response: The CES-D is a well-validated screening tool in large HIV cohorts, including the legacy MACS and WIHS. We used the established cut-off score of >= 16 to indicate elevated depressive symptoms. We added references to the revised manuscript supporting its reliability in this specific population.

**6. The selected sample is not the entire MWCCS; presumably, there was missingness of key variables. How did the selection bias affect the results?**

Response: Our final analytic sample included 1,622 PLWH and 908 PLWoH who had complete data for the primary variables at the baseline visit. To improve transparency regarding exclusions, we added a participant flow diagram (Figure 1) detailing the number of participants at each visit, the construction of the analytic datasets, and exclusions due to missing eGFR. We also expanded the Limitations section to acknowledge potential selection bias due to requiring non-missing eGFR and key measures.
 **Minor
1. The text is sparsely referenced, yet there is a plethora of literature about HIV, CKD, nephrotoxicity, and depression.**

Response: We have thoroughly reviewed and updated the reference and text in the literature.

**2. Please use consistent word tense; in some places past tense is used and in others present tense.**

Response: We have performed a thorough review to ensure the manuscript consistently uses the past tense for methods and results and the present tense for established facts.

**3. What measures of kidney function indicated worse outcomes among the PLWH ? The outcomes are not stated in the methods.**

Response: We have clarified that kidney function was assessed using eGFR (CKD-EPI equation) and categorized into six stages.

**4. Please clarify what is meant by "other individuals" in the statement "Among marital categories,
widowed and “other” individuals exhibited elevated CKD rates" found in the first paragraph of page 9.
In the same page, please clarify what you meant by "non-Hispanic Other individuals".**

Response: We have updated in the methods. "Other" marital status includes categories outside of married, widowed, divorced, separated, or never married. "Non-Hispanic Other" race includes individuals who do not identify as White, Black, or Hispanic.

**Reviewer 2**

**1. Clarification of GEE model specification and adjustment set**

**Response:** We have added additional univariate analysis in Table 4 results. The revised Table 4 presents univariate repeated-measures GEE models (one predictor at a time) using Visits 101-103, a Poisson distribution with log link, an exchangeable working correlation structure, and robust variance estimates. To avoid Table 2-style misinterpretation, we do not present a single multivariable “Table 2” model; accordingly, we describe these GEE results as associations rather than causal effects.

**2. Mediation analysis: Potential over adjustment and model transparency**

The mediation analysis requires clearer description. It is not evident whether depressive symptoms and nephrotoxic medication use were included as covariates in the primary GEE model. If mediators were adjusted for in the outcome model used to estimate the total effect, this would bias the estimated direct effect of HIV on CKD and undermine the validity of the mediation analysis. Standard counterfactual mediation approaches require that mediators not be controlled for in the total effect model.

The authors should explicitly state:

•	Which covariates were included in the GEE model estimating the total effect

•	Which covariates were included in the mediator and outcome models

•	Whether any variables on the causal pathway were inadvertently adjusted for

Including a DAG or a brief justification of the adjustment set would strengthen the causal interpretation.

**Response:** We have revised the manuscript to explicitly clarify the modeling strategy and to ensure that no mediators were included in the total-effect model.

To avoid overadjustment bias, depressive symptoms and nephrotoxic medication use were not included in the total-effect model estimating the association between HIV status and CKD. This total-effect model adjusted for the prespecified baseline confounder set (C): age, sex at birth, race/ethnicity, marital status, employment status, diabetes, hypertension, current smoking, binge drinking, marijuana use, cocaine use, heroin use, other drug use, and IDU. The total-effect model used for mediation is distinct from the univariate GEE models presented in Table 4 and was estimated within the counterfactual mediation framework. No mediators were included in the total-effect model.

For the mediation analysis, we used a counterfactual framework (PROC CAUSALMED). The mediator model (HIV → mediator) and the outcome model (HIV + mediator → CKD) included the same baseline confounder set (C) described above. This approach allowed us to estimate the total effect (TE), natural direct effect (NDE), and natural indirect effect (NIE) while appropriately separating direct and mediated pathways. Exposure-mediator interaction was permitted in the decomposition. To further improve transparency, we included a prespecified DAG (Figure 2) illustrating the assumed causal structure and the rationale for the adjustment set.

**3. Construction and heterogeneity of the nephrotoxic medication exposure**

The definition of nephrotoxic medication exposure requires more detail. It is unclear whether the exposure reflects any use, cumulative use, dose, duration, or specific drug classes. Nephrotoxic medications are heterogeneous, and collapsing them into a single binary indicator risks substantial exposure misclassification. This is particularly important because the mediation analysis treats this variable as a mechanistic pathway.

The authors should consider:

•	Describing how the exposure was constructed

•	Justifying the chosen categorization

•	Considering sensitivity analyses using alternative definitions (e.g., cumulative burden, class specific indicators)

•	Discussing the implications of heterogeneity within the exposure

**Response:** We defined exposure as a binary indicator (visit-level indicator reflecting any recorded use of ≥1 nephrotoxic medication at that visit’s medication inventory; dose, duration, and cumulative burden were not harmonized and therefore were not modeled in the primary analysis), based on expert review. We acknowledge that drug classes differ in toxicity. Per your suggestion, we have added a sensitivity analysis in the supplement breaking down the risk by specific drug classes where sample size permits. We also expanded the Discussion to emphasize the possibility of confounding by indication and reverse causality for classes commonly prescribed in response to kidney risk (e.g., ACEi/ARB, diuretics).

**4. Interpretation of depression findings and internal inconsistency**

The discussion presents conflicting explanations for the null association between depression and CKD. On one hand, the authors suggest that effective depression treatment in the cohort may explain the lack of association. On the other hand, they acknowledge that depression was defined solely by CES D scores, without information on diagnosis or treatment, making it impossible to determine whether participants were receiving appropriate care.

These two statements are contradictory. Moreover, part of the hypothesized link between depression and CKD involves the use of nephrotoxic antidepressants, which complicates the interpretation of “effective treatment” as a protective factor.

The authors should consider reconciling these points and provide a more coherent interpretation of the null finding.

**Response:** We have provided more information in the Discussion. We removed speculative statements implying “effective treatment” as an explanation because we lack clinical diagnosis and treatment data. We now interpret the null finding more cautiously, noting that depression was defined solely based on CES-D symptom scores without information on diagnosis, treatment, adherence, or response. We have also addressed the complexity of nephrotoxic antidepressants as a potential confounder and the possibility of confounding by indication.

**5. Abstract should explicitly state the null finding for depression**

Given that depression is central to the study’s rationale, title, and keywords, the abstract should explicitly state that depressive symptoms were not associated with CKD in the adjusted analyses and did not mediate the HIV-CKD relationship. This omission may mislead readers and should be corrected.

**Response**: We have revised the Abstract to explicitly state that depressive symptoms were not associated with CKD in adjusted analyses and did not mediate the HIV-CKD relationship. We also clarified that the repeated-measures GEE results are based on univariate models.

Moderate Comments

**6. Flow chart of recruitment and analytic sample**

The description of recruitment, retention, and analytic sample construction is complex. A flow chart would greatly improve clarity by illustrating:

•	the number of participants at each visit

•	how many were new vs. returning

•	how many were included in each analysis

•	reasons for exclusion

This would help readers understand the analytic dataset.

**Response**: We have added a flow chart illustrating participant enrollment, retention across visits (101-103), and reasons for exclusion. This is presented as Figure 1 (Study Flow Chart), and the causal pathway diagram is presented as Figure 2.

**7. Organization of the Discussion**

The discussion would benefit from beginning with a concise summary of the study’s aims and key findings before moving into interpretation. This would help orient readers and improve flow.

Additionally, the section discussing the etiology of nephropathy in the pre ART era seems more appropriate for the introduction section, where it would better contextualize the biological plausibility of HIV related kidney injury.

**Response**: We have updated the Discussion to begin with a concise summary of study aims and key findings to improve flow and orientation. The historical section on pre-ART HIVAN has been moved to the Introduction.

**Minor Comments**

There is an incomplete sentence in the discussion:

“Studies such as those by Capuron et al. and Lustman et al. have suggested that while somatic depressive symptoms (e.g., fatigue, appetite loss) are more prevalent in conditions like diabetes, cognitive symptoms (e.g., sadness, hopelessness) may be more prominent in populations of persons with [12, 32].”

**Response**: The sentence on page 20 regarding "cognitive symptoms" has been corrected to ensure it is complete.

**Overall Assessment**

This manuscript addresses an important topic and has the potential to make a meaningful contribution. Clarifying the analytic methods, improving internal consistency, and strengthening the organization would substantially enhance the rigor and interpretability of the findings.

**Response**: We thank reviewers for their comments and suggestions.
